# Supplementary material for: Citizen science for predicting spatio-temporal patterns in seabird abundance during migration
Source: PLoS One. 2020 Aug 14;15(8):e0236631. doi: 10.1371/journal.pone.0236631 (PMC7428152; doi:10.1371/journal.pone.0236631)
Supplement: S1 File — (DOCX) [file pone.0236631.s001.docx]

**Citizen science for predicting spatio-temporal patterns in seabird abundance during migration**

Beatriz Martín, Alejandro Onrubia, Julio González-Arias, Juan A. Vicente-Vírseda

**Supplementary Methods:** **Detailed description of the set of predictors included in the models**

As migrating birds need to replenish energy reserves during stopover periods at key locations where maximize their refueling opportunities [1,2], fluctuations in bird abundance during migration are closely related to changes in food resources [1]. Therefore, most of the variation in seabirds appears to be mediated by changes in prey abundance [2,3]. We used chlorophyll concentration (Chla, measured in mg.m-3) as a proxy of marine productivity [4]. Specifically, we downloaded satellite-based monthly products at 4 km spatial resolution for 2003-2017 period (JRC Data Catalogue; <http://gmis.jrc.ec.europa.eu/satellite/4km/>). Discard availability may influence the at-sea distribution of shearwaters [5,6]. As a proxy of food availability (both discards and fishes) we used information on fisheries. This information was inferred from the distribution of fishing vessels [7] between 2014 and 2015, sourced by the JRC Data Catalogue at 1 km resolution (<http://gmis.jrc.ec.europa.eu/dataset/jrc-fad-ais1415>). This product identifies which are the areas where fishing is more frequent. Vessel tracking data was derived from the Automatic Identification System (AIS), an open source data allowing to analyse the relation between fishing communities and fishing areas at high spatial resolution across Europe. Specifically, data used to build the map consist of 150 million positions from European fishing vessels above 15 m in length. In spite of its limited temporal coverage, the main strength of this dataset is its fine spatial resolution. This proxy on food availability in spatial terms, however, is complemented in our analysis with the high temporal resolution in marine productivity provided by chlorophyll concentration.

Together with chlorophyll concentration, sea surface temperature (SST) is also a proxy of water mass distributions, frontal systems, and ocean productivity [8–10]. In shearwaters, which perform dynamic soaring flight, oceanic winds are also of main importance in modulating migratory behavior [11]. Wind speed and direction usually affect both migratory behavior [12,13] (but see [14]) and seabird detectability by the observers [15]. Data on SST and wind were obtained through RNCEP package [16] in R [17] which allowed to request data from the NCEP/NCAR Reanalysis dataset (NOAA ESRL Physical Sciences Division; https://www.esrl.noaa.gov/psd/data/gridded/data.ncep.reanalysis2.html) for a specified range of space and time based on the observations of shearwaters. Specifically, we requested daily values at midday at the surface level, since Balearic shearwaters tend to migrate at very low height [15]. Variables requested were “air.995” (i.e. air temperature), “uwind.sig995” (i.e., u-wind component) and “vwind.995” (i.e. v-wind component). RNCEP interpolates the nearest data value (in terms of location and time) in the NCEP/NCAR dataset to provide the requested information. We also considered the standard deviation (associated standard deviation of the points used to perform the interpolation) of the meteorological values (i.e., u-wind, v-wind and temperature) used for this interpolation as additional predictors in our models (Table 1). General flight activity of seabirds increases during moonlit nights and moon phase has been shown to affect shearwater migration behaviour [18]. As changes in the flying patterns during night may also affect daytime flights, we considered the daily fraction of the moon illuminated at midnight as an additional predictor, which was sourced by the U.S Naval Observatory & Astronomical Applications Department (<http://aa.usno.navy.mil/data/>).

Together with the usual interannual variability in food resources, long-term climate change may also affects the at-sea distribution of this species [1,19,20] likely through effects on fish stocks [21]. The possible impact of climate change in the distribution of shearwaters at a regional level was taken into account through the North Atlantic Oscillation Index (NAO; [22]) obtained from the Climate Prediction Center (US National Weather Service, NOAA), as monthly data from 1950 to 2018 (<http://www.cpc.ncep.noaa.gov/products/precip/CWlink/pna/nao.shtml>). As SST and wind predictors already provided a daily resolution of the weather conditions at specific locations, monthly values rather than daily NAO index were preferred, as a surrogate of regional climate conditions affecting shearwater migration at a more general and longer timeframe.

Water depth has been shown to influence seabird distributions [23]. Bathymetry was used as a surrogate of coastal-pelagic areas (Afán et al., 2019). Bathymetric data were sourced by European Marine Observation and Data Network (EMODnet; <http://www.emodnet-bathymetry.eu/data-products>) at ~200 m resolution although they were later aggregated through a moving-average window to 10 km resolution in order to obtain a more general bathymetry pattern regarding the shearwater observation location. This cell size was selected as a compromise between this general pattern in bathymetry and the sufficient resolution for conservation purposes.

**Supplementary Results:**


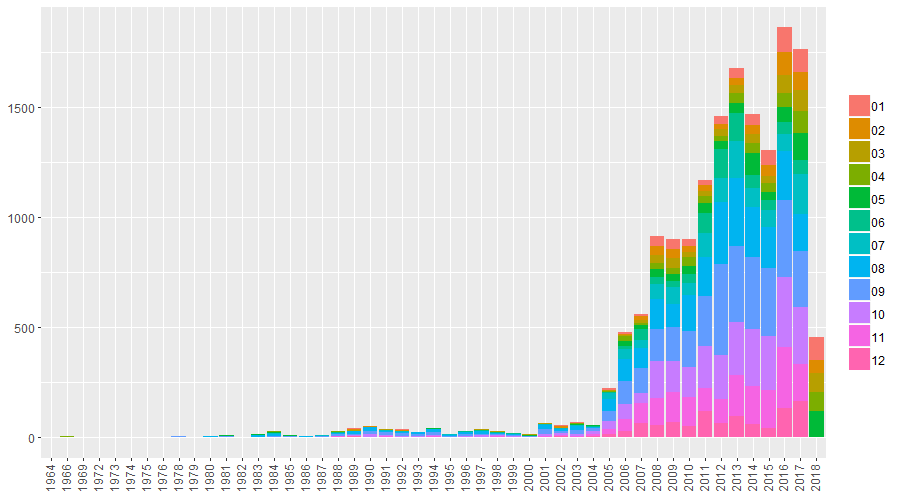


Fig. S1. Exploratory analysis of the data on shearwater abundance. Monthly annual number of records between 1964-2018. Months (from January -1- to December -12- are indicated as different colours along the bars of the plot.


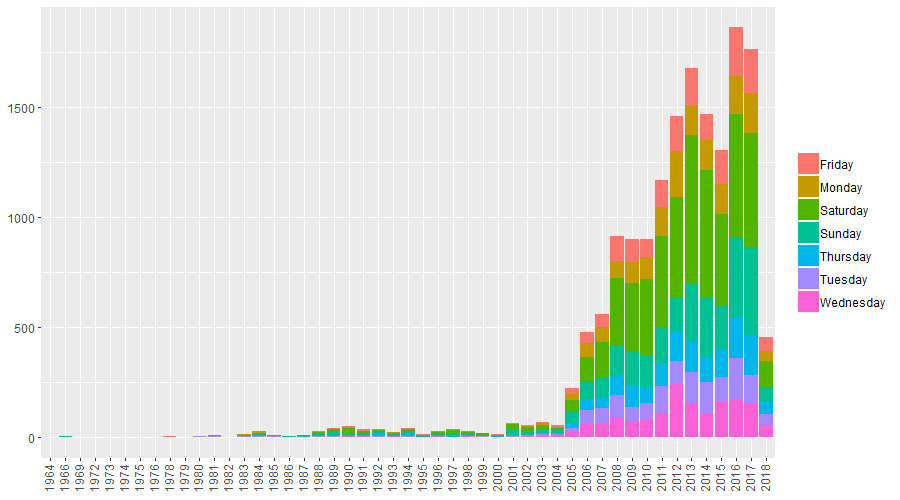


Fig. S2. Exploratory analysis of the data on shearwater abundance. Annual number of records per day of the week between 1964-2018. Days of the week are indicated as different colours along the bars of the plot.


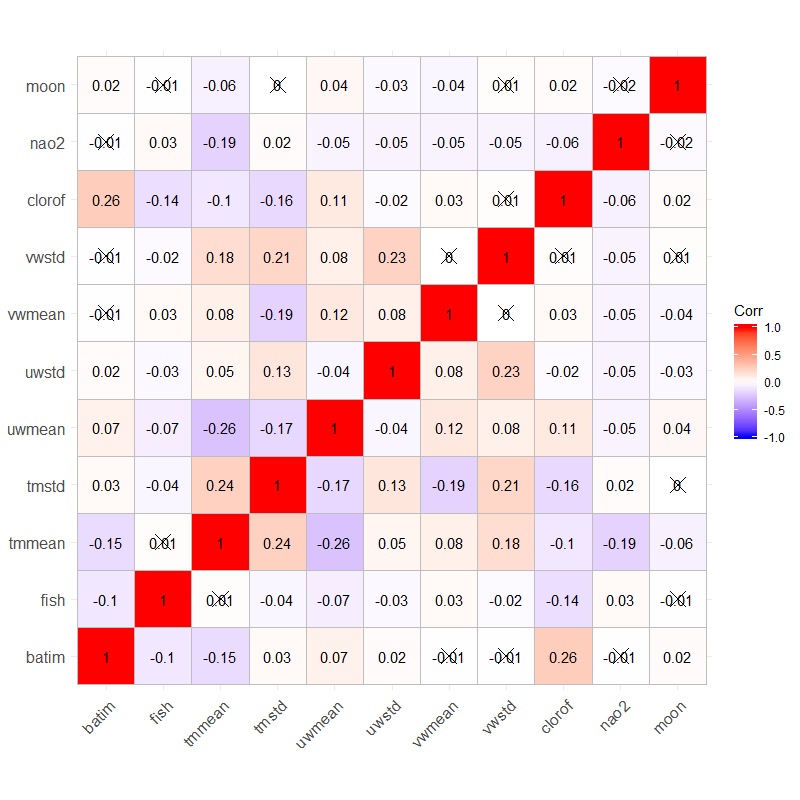


Fig. S3. Assessment of collinearity. Pearson correlation coefficients among pairs of predictors. Non significant results at the 0.05 p-level are crossed out.

a)
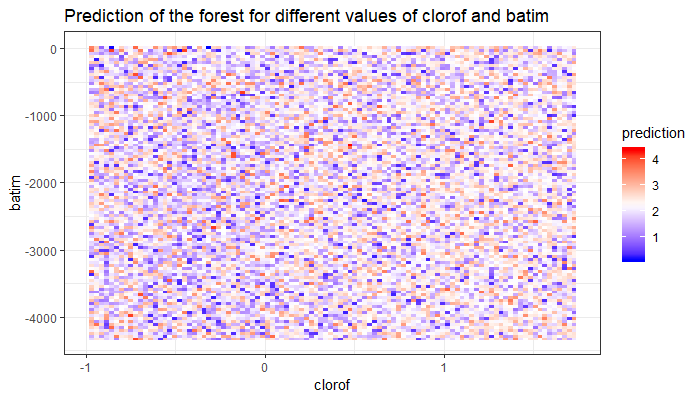


b)


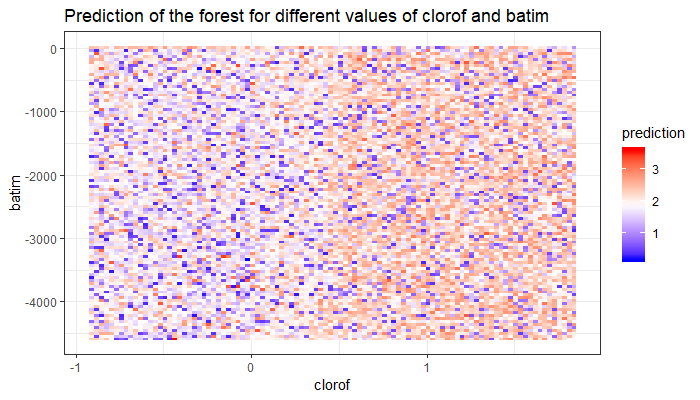


Fig. S4. Interactions between variables. Predicted abundance of shearwaters (pedictions in the natural logarithmic scale) for different values of chlorophyll and bathymetry. (a) pre-breeding; (b) post-breeding migration models.

a)


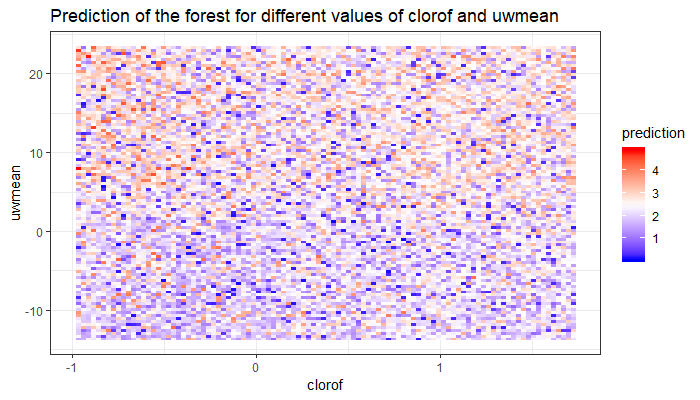


b)


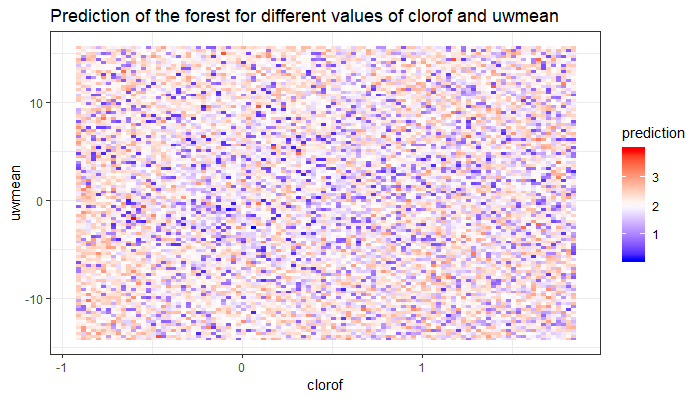


Fig. S5. Interactions between variables. Predicted abundance of shearwaters (pedictions in the natural logarithmic scale) for different values of chlorophyll and u-wind component. (a) pre-breeding; (b) post-breeding migration models.

a)


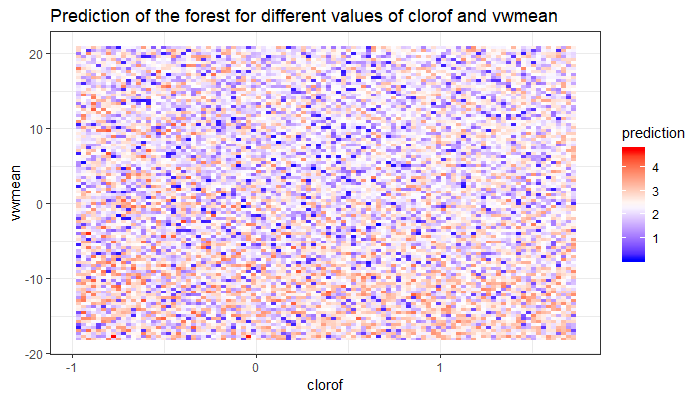


b)


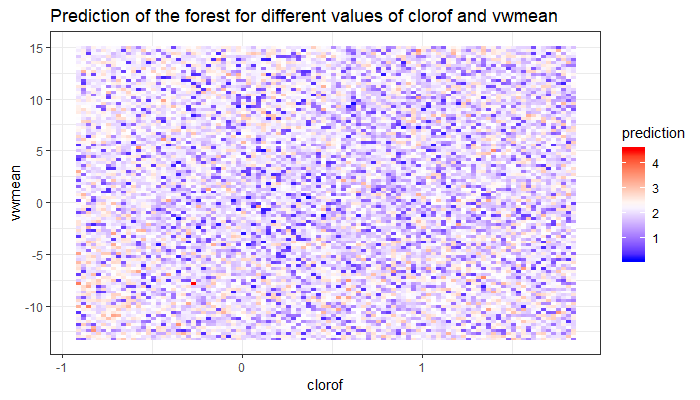


Fig. S6. Interactions between variables. Predicted abundance of shearwaters (pedictions in the natural logarithmic scale) for different values of chlorophyll and v-wind component. (a) pre-breeding; (b) post-breeding migration models.

**Supplementary references:**

1. Wynn RB, Josey S, Martin A, Johns D, Yésou P. Climate-driven range expansion of a critically endangered top predator in northeast Atlantic waters. Biology Letters. 2007;3: 529–532. doi:10.1098/rsbl.2007.0162

2. Benoit-Bird KJ, Battaile BC, Heppell SA, Hoover B, Irons D, Jones N, et al. Prey Patch Patterns Predict Habitat Use by Top Marine Predators with Diverse Foraging Strategies. PLOS ONE. 2013;8: e53348. doi:10.1371/journal.pone.0053348

3. Frederiksen M, Edwards M, Richardson AJ, Halliday NC, Wanless S. From plankton to top predators: bottom-up control of a marine food web across four trophic levels. Journal of Animal Ecology. 2006;75: 1259–1268. doi:10.1111/j.1365-2656.2006.01148.x

4. Wakefield E, Phillips R, Matthiopoulos J. Quantifying habitat use and preferences of pelagic seabirds using individual movement data: A review. Marine Ecology-progress Series - MAR ECOL-PROGR SER. 2009;393. doi:10.3354/meps08203

5. Cortés V, García-Barcelona S, González-Solís J. Sex- and age-biased mortality of three shearwater species in longline fisheries of the Mediterranean. Marine Ecological Progress Series. 2018;588: 229–241.

6. Genovart M, Bécares J, Igual J-M, Martínez-Abraín A, Escandell R, Sánchez A, et al. Differential adult survival at close seabird colonies: The importance of spatial foraging segregation and bycatch risk during the breeding season. Global Change Biology. 2018;24: 1279–1290. doi:10.1111/gcb.13997

7. Natale F, Gibin M, Alessandrini A, Vespe M, Paulrud A. Mapping Fishing Effort through AIS Data. PLOS ONE. 2015;10: e0130746. doi:10.1371/journal.pone.0130746

8. Afán I, Navarro J, Cardador L, Ramirez F, Kato A, Rodríguez B, et al. Foraging movements and habitat niche of two closely related seabirds breeding in sympatry. Marine Biology. 2014;161: 657–668. doi:10.1007/s00227-013-2368-4

9. Ramos R, Granadeiro JP, Nevoux M, Mougin J-L, Dias MP, Catry P. Combined Spatio-Temporal Impacts of Climate and Longline Fisheries on the Survival of a Trans-Equatorial Marine Migrant. PLOS ONE. 2012;7: e40822. doi:10.1371/journal.pone.0040822

10. Robinson J, Dornelas M, Ojanguren AF. Interspecific synchrony of seabird population growth rate and breeding success. Ecology and evolution. 2013;3: 2013–9. doi:10.1002/ece3.592

11. González-Solís J, Felicisimo A, Fox J, Afanasyev V, Kolbeinsson Y, Muñoz J. Influence of sea surface winds on shearwater migration detours. Marine Ecology-progress Series - MAR ECOL-PROGR SER. 2009;391: 221–230. doi:10.3354/meps08128

12. Dias MP, Granadeiro JP, Catry P. Do seabirds differ from other migrants in their travel arrangements? On route strategies of Cory’s shearwater during its trans-equatorial journey. PLoS One. 2012;7: e49376–e49376. doi:10.1371/journal.pone.0049376

13. Catry P, Dias MP, Phillips RA, Granadeiro JP. Different Means to the Same End: Long-Distance Migrant Seabirds from Two Colonies Differ in Behaviour, Despite Common Wintering Grounds. PLOS ONE. 2011;6: e26079. doi:10.1371/journal.pone.0026079

14. Dell’Ariccia G, Benhamou S, Dias MP, Granadeiro JP, Sudre J, Catry P, et al. Flexible migratory choices of Cory’s shearwaters are not driven by shifts in prevailing air currents. Scientific Reports. 2018;8: 3376. doi:10.1038/s41598-018-21608-2

15. Martín B, Onrubia A, Ferrer M. Endemic shearwaters are increasing in the Mediterranean in relation to factors that are closely related to human activities. Global Ecology and Conservation. 2019;in press: e00740.

16. Kemp MU, Emiel van Loon E, Shamoun-Baranes J, Bouten W. RNCEP: global weather and climate data at your fingertips. Methods in Ecology and Evolution. 2012;3: 65–70. doi:10.1111/j.2041-210X.2011.00138.x

17. R Development Core Team. R: A language and environment for statistical computing. R Foundation for Statistical Computing. 2018.

18. Dias MP, Granadeiro JP, Catry P. Do Seabirds Differ from Other Migrants in Their Travel Arrangements? On Route Strategies of Cory’s Shearwater during Its Trans-Equatorial Journey. PLOS ONE. 2012;7: e49376. doi:10.1371/journal.pone.0049376

19. Luczak C, Beaugrand G, Jaffré M, Lenoir S. Climate change impact on Balearic shearwater through a trophic cascade. Biology Letters. 2011;7: 702–705. doi:10.1098/rsbl.2011.0225

20. Votier SC, Bearhop S, Attrill MJ, Oro D. Is climate change the most likely driver of range expansion for a critically endangered top predator in northeast Atlantic waters? Biology Letters. 2008;4: 204–205. doi:10.1098/rsbl.2007.0558

21. Tsikliras AC, Licandro P, Pardalou A, McQuinn IH, Gröger JP, Alheit J. Synchronization of Mediterranean pelagic fish populations with the North Atlantic climate variability. Deep Sea Research Part II: Topical Studies in Oceanography. 2019;159: 143–151. doi:10.1016/j.dsr2.2018.07.005

22. Visbeck MH, Hurrell JW, Polvani L, Cullen HM. The North Atlantic Oscillation: Past, present, and future. Proc Natl Acad Sci USA. 2001;98: 12876. doi:10.1073/pnas.231391598

23. Yen PPW, Sydeman WJ, Hyrenbach KD. Marine bird and cetacean associations with bathymetric habitats and shallow-water topographies: implications for trophic transfer and conservation. Journal of Marine Systems. 2004;50: 79–99. doi:10.1016/j.jmarsys.2003.09.015

24. Afán I, Navarro J, Grémillet D, Coll M, Forero M. Maiden voyage into death: are fisheries affecting seabird juvenile survival during the first days at-sea? Royal Society Open Science. 31 Jan 20196: 181151. doi:10.1098/rsos.181151
