# Supplementary material for: Citizen science for predicting spatio-temporal patterns in seabird abundance during migration
Source: PLoS One. 2020 Aug 14;15(8):e0236631. doi: 10.1371/journal.pone.0236631 (PMC7428152; doi:10.1371/journal.pone.0236631)
Supplement: Attachment — Submitted filename: Balearic_shearwater_migration_eBird_sep19_PLOSONE_kdh.docx [file pone.0236631.s002.docx]

**Citizen science and machine learning for predicting spatio-temporal patterns in seabird migration**

Beatriz Martín ^1*¶^, Alejandro Onrubia ^1¶^, Julio González-Arias^2&^, Juan A. Vicente-Vírseda^2&^

^1^ Fundación Migres, CIMA, Tarifa, Spain

^2^ Business and Finance Department, Faculty of Economics and Business, UNED, Madrid, Spain

^*^ Corresponding author: Beatriz Martín

E-mail: [bmartin@fundacionmigres.org](mailto:beatrizmartin@ebd.csic.es)

^¶^These authors contributed equally to this work.

^&^These authors also contributed equally to this work.

**Abstract**

Here we undertook the first long-term study on the distribution of migrating shearwaters from data gathered within the framework of citizen science projects. Specifically, we collected daily abundance of Balearic shearwaters from 1964 to 2018 from the online databases Trektellen and eBird. We applied machine-learning techniques, specifically Random Forest models, to predict shearwater abundance during migration using 15 environmental predictors. We built separated models for pre-breeding and post-breeding migration. When evaluated for the total data sample, the models explained more than 52% of the variation in shearwater abundance. The models also showed good ability to predict shearwater distributions for both migration periods (correlation between observed and predicted abundance was about 70%). However, relative variable importance and variation among the models built with different training data subsamples differed between migration periods. Our results showed that data gathered in citizen science initiatives together with recently available high-resolution satellite imagery, can be successfully applied to describe the migratory spatio-temporal patterns of seabird species accurately. We show that a predictive modelling approach may offer a powerful and cost-effective tool for the long-term monitoring of the migratory patterns in sensitive marine species, as well as to identify at sea areas relevant for their protection. Modelling approaches can also be essential tools to detect the impacts of climate and other global changes in this and other species.

**Introduction**

Seabirds are top predators at the end of the aquatic trophic chain thus they can be indicators on a global scale of the status of pelagic ecosystems and the changes therein [1]. In addition, seabirds are one of the most threatened groups of marine vertebrates, partly because they are highly mobile and migrate, thus they are subjected to many different environmental conditions over the year [2]. Studies on pelagic seabirds, however, have been traditionally focused on the breeding and wintering areas [3], whereas their long-distance migratory movements have remained poorly understood until recent tracking technology development [4]. Therefore, there is still a gap in the knowledge on migration and at sea distribution in most of these species [5]. During migration, birds spend relatively little time in any one place compared with other seasons [6]. Despite the short duration of the stay, stopovers are key sites, since low resource availability or disturbances ~~at stopovers~~ can reduce the survival of the individuals and limit the population size [7].Therefore, conservation efforts should address not only wintering and breeding grounds, but the en-route locations during migration [6].

However, determining the spatial distribution of seabirds during the migration period is a difficult task, because surveys ~~of birds~~ are usually limited to specific subsets of the total migratory route [8–11]. Ringing recoveries are useful for identifying very general movement patterns, but they are not enough to determine stopover areas, which are of main importance for understanding the migratory patterns as well as to inform conservation planning at sea [12]. The continuous technical development of electronic devices for tracking animal movements ~~(such as geolocators, accelerometers, GPSs, and PTT devices)~~ have contributed to the knowledge on the at-sea distribution of many seabird species [13,14]. However, costs limit the sample size in studies using electronic devices, even with the most expensive budgets [15]. If sample size is limited, tracking data may not represent the full species migratory range due to colony- and individual specific migratory strategies [16], thus important stopover areas and foraging grounds for a given species may not be identified. This is particularly true for long-lived ~~bird~~ species, such as many seabirds, which have a great capacity to alter migratory behavior in response to environmental variability (e.g., Martín et al., 2014; Scholer et al., 2016) and in relation to specific individual traits (e.g., Afán et al., 2019; Guilford et al., 2012). Moreover, limited duration of electronic devices usually does not allow for long-term monitoring of the impacts of climate. Therefore, these devices, even providing very detailed information on the seabird movements, have a limited ability to improve our understanding of the adaptation of migration strategies to deal with a changing environment at both population and species levels [9,21]. Therefore, it is crucial to develop alternative methods, particularly for the most endangered species, to undertake the long-term monitoring of their at sea locations, including stopover sites [5].

In this sense, the active public involvement in scientific research (i.e., citizen science) has become a key source of high-quality data for scientists and policymakers [22]. Among others, citizen science projects have enabled researchers to obtain a comprehensive picture of habitat use in many different species [23]. These citizen science projects provide millions of species observations each year [24,25]. As compared with the detailed data gathered from electronic devices, the massive datasets from citizen science projects have the advantage of offering low-cost information on long-term temporal and large spatial extents from many different individuals belonging to several populations (e.g., [26]. Together with citizen science projects, there is an increasing availability of large-scale environmental data (e.g., satellite imagery), which can be used ~~as environmental variables~~ to predict species distributions over large areas. Although these large volumes of data maybe hard to analyze and interpret, and relevant interpretations may be limited in scope, accurate information on the distribution of species during the entire annual cycle can be derived from these datasets, even for populations of long-distance migratory bird species [27].

The Balearic shearwater (*Puffinus mauretanicus*) only breeds in the Balearic Islands (Spain) and it is considered as one of the most threatened seabirds in the world [28]. Balearic shearwaters, spend about one quarter of the year on migration [20]. To date, the ~~main~~ existing scientific knowledge on the Balearic shearwater migratory ~~spatial patterns~~ is based on TWO STUDIES USING GEOLOCATOR ARCHIVAL TAGS: (i) 26 individuals FROM ~~breeding at a~~ THE large breeding colony in Mallorca, tracked ~~using geolocators~~ between 2010-2011 [20], ~~together with~~ data (ii) ~~from~~ 16 individuals breeding in Eivissa Island ~~(western Mediterranean)~~ monitored during ~~the~~ 2011-2012 ~~period based on light level geoloCators~~ [21]. Additional surveys using ship transects along the coast of the western Iberian Peninsula from 2004 to 2009 [10], and land- and boat-based surveys in 2007-2010 [9] have also provided partial information on the at-sea distribution of this species during the non-breeding season. However, unused areas according to these studies may be relevant for other Balearic shearwater populations. ~~Moreover~~, YET, these studies showed that areas used by shearwaters may change from year to year in relation to interannual variability in the environmental conditions [9,20], as well as among different breeding colonies [21]. MOREOVER, unused areas according to these studies may be relevant for other Balearic shearwater populations

During migration, Balearic shearwaters occur in relatively shallow, coastal waters along the shoreline, allowing their observation from land-based sites [8,29]. Therefore, citizen science projects recording Balearic shearwaters from the coast may provide a useful ~~data~~ RECORDS for the MONITORING ~~long-term monitoring of~~ this species along its migratory route, completing the partial picture offered by geolocators [20,21] and vessel surveys [9,10]. Modelling techniques ~~based on these datasets~~ can be USED ~~applied to~~ predict the distribution of the Balearic shearwaters along the coast, ~~in order~~ to identify the most likely pelagic habitats used during migration, both over time and across space (Guisan and Zimmermann, 2000).

The aim of this study was to determine the performance of data gathered ~~within~~ FROM citizen science projects to predict the spatial distribution of migrating seabirds in order to identify marine important areas for the conservation of these and other species. Specifically, we undertook a multi-year study of the spatio-temporal patterns in Balearic shearwater migration using data gathered within eBird [27] and Trektellen [31], two biodiversity-related citizen science projects gathering records of birds provided by professional and amateur ornithologists around the world. Making use of these datasets, we provide insights into the spatial distribution and the variation (seasonal and interannual) of the migratory patterns of this species. Particularly, we aimed to: (1) determine general regional environmental predictors driving spatial use at sea during migration, (2) assess whether these variables differ between pre-breeding and post-breeding migration, AND (3) ~~to~~ determine possible trends in the migratory spatial patterns by identifying latitudinal changes along the migration route in relation to ~~climate change~~ ENVIRONMENTAL VARIABLES~~.~~

**Materials and methods**

**Study area**

The extent of the study area (Fig 1) covers the whole distribution range of the Balearic shearwater throughout the year [28].

**Fig. 1.** **Balearic shearwater observations.** Spatial distribution of observations of Balearic shearwaters considered in the analysis. a) pre-breeding migration (n=7,492); b) post-breeding migration (n=4,510). Period 2005-2017.

## Study species

The Baleraric shearwater (*Puffinus mauretanicus*) is included as ‘Critically Endangered’ on the IUCN Red List (BirdLife International, 2018) and ~~it~~ is also considered a~~s~~ threatened bird for the European Union (i.e., rare or vulnerable bird species as listed in Annex I of the E.U. Bird Directive). Most of the population of Balearic shearwater leaves the Mediterranean each year after breeding [20,32] and mostly remains in the Atlantic during the non-breeding season. At sea, it usually occurs in productive shelf areas related to oceanographic frontal systems [10,21]. During migration, Balearic shearwaters tend to fly very close to the shoreline [8]. Fluctuations in Balearic shearwater migration, both seasonally and INTER-annually, seem to be related to changes in food resources [33]. Balearic shearwater’s diet includes small pelagic and demersal fish, frequently obtained from trawling discards, also INCLUDES plankton and macrozooplankton, specifically krill [34,35].

**Model variables**

**Abundance of shearwaters**

Daily abundance of the species was recorded from 1964 to 2018, both as opportunistic sighting records and within systematic effort-based surveys obtained from the online databases Trektellen [31] and eBird [36]. Due to the opportunistic nature of some of these data, we needed powerful modelling techniques to obtain robust results (see below). Although it was possible to differentiate opportunistic and systematic surveys within the dataset, we opted for keeping all records for our analysis in order to test the robustness of the modelling approach in case this information was not available for one particular species. Since some records contained in Trektellen database may be also included in eBird, based on date and spatial location, we avoid duplicated observations between databases. From Trektellen database, according to the migratory range of the study species, we specifically obtained observations from 123 observatories in France, Portugal, Spain and UK, from 2005 to 2018, counting 7,619 different observations and totaling 378,112 birds. Regarding eBird records, we analysed data on 258,730 birds observed in 8,244 different records from 1964 to 2018. From this total sample of birds, we only modelled data collected during the migration period. We defined migratory periods based on the known phenology of the Balearic shearwater. Specifically, we considered ‘post-breeding migration’, the northward migration of birds leaving the Mediterranean and molt, between May – August, and ‘pre-breeding migration’, corresponding to the southward migration of birds from the Atlantic and ~~coasts~~ returning to the breeding areas in the Mediterranean, from September to December [37,38]. Birds at their breeding grounds in the Balearic Islands (i.e., birds observed at land) were removed from the data sample.

**Environmental predictors**

To model Balearic shearwater abundance, expressed as the number of birds sighted on a given date at a given latitude / longitude, during migration we used 15 environmental predictors (Table 1) that have been previously described to be related with the spatial distribution at sea of this and other seabird species [10,19,39,39–45].

**Table 1.** **Variable description.**

| **Name** | **Description** | **unit** | **Source** | **Period** |
| --- | --- | --- | --- | --- |
| **Totals** | shearwater abundance | number of birds | Trektellen; eBird | 2005-2018; 1964-2018 |
| **XLONG** | longitude | degrees | Trektellen; eBird | 2005-2018; 1964-2018 |
| **YLAT** | latitude | degrees | Trektellen; eBird | 2005-2018; 1964-2018 |
| **batim** | bathymetry | meters | EMODnet (*European Marine Observation and Data Network*) | 2012 |
| **fish** | fishing intensity | number of vessels | JRC Data Catalogue; “Automatic Identification System” (AIS) | 2014-2015 |
| **tmmean** | mean temperature | º K | NCEP/NCAR Reanalysis dataset (NOAA ESRL Physical Sciences Division) | 1964-2018 |
| **tmstd** | standard deviation of mean temperature | º K | NCEP/NCAR Reanalysis dataset (NOAA ESRL Physical Sciences Division) | 1964-2018 |
| **uwmean** | mean wind speed (u-wind: east-west direction) | m/s | NCEP/NCAR Reanalysis dataset (NOAA ESRL Physical Sciences Division) | 1964-2018 |
| **uwstd** | standard deviation of mean wind speed (u-wind: east-west direction) | m/s | NCEP/NCAR Reanalysis dataset (NOAA ESRL Physical Sciences Division) | 1964-2018 |
| **vwmean** | mean wind speed (v-wind: north-south direction) | m/s | NCEP/NCAR Reanalysis dataset (NOAA ESRL Physical Sciences Division) | 1964-2018 |
| **vwstd** | standard deviation of mean wind speed (v-wind: north-south direction) | m/s | NCEP/NCAR Reanalysis dataset (NOAA ESRL Physical Sciences Division) | 1964-2018 |
| **clorof** | chlorophyll concen~~cen~~tration | mg m-3 | JRC Data Catalogue; MERIS | 2004-2017 |
| **NAO** | NAO index | - | Climate Prediction Center (US National Weather Service, NOAA) | 1950-2018 |
| **moon** | daily fraction of the moon illuminated at midnight | % | U.S Naval Observatory & Astronomical Applications Department | 2005-2018 |
| **Julian** | julian date | date of the observation | Trektellen; eBird | 2005-2018; 1964-2018 |
| **Year** | year | year of the observation | Trektellen; eBird | 2005-2018; 1964-2018 |

Description of the set of predictors included in the models.

As migrating birds need to replenish energy reserves during stopover periods at key locations where maximize their refueling opportunities [46], fluctuations in migration are closely related to changes in food resources [47]. Therefore, most of the variation in seabirds appears to be mediated by changes in prey abundance [48,49]. Although precise data on food availability and their year-to-year variation are hard to obtain in natural conditions, we included in our models two proxies of food availability for shearwaters: phytoplankton biomass and fisheries. Specifically, we used algal biomass (chlorophyll concentration, Chla in mg.m-3) as a proxy for phytoplankton biomass [50]. We obtained chlorophyll concentration as the product from satellite-based optical sensors monthly available at 4 km resolution for 2003-2017 period (JRC Data Catalogue; <http://gmis.jrc.ec.europa.eu/satellite/4km/>). Discard availability may influence the at-sea distribution of shearwaters [51,52]. Information on fisheries was inferred from the distribution of fishing vessels [53] between 2014 and 2015, sourced by the JRC Data Catalogue at 1 km resolution (<http://gmis.jrc.ec.europa.eu/dataset/jrc-fad-ais1415>).

Together with chlorophyll concentration, sea surface temperature (SST) is also a proxy of water mass distributions, frontal systems, and ocean productivity [39,44,45]. In shearwaters, which perform dynamic soaring flight, oceanic winds are also of main importance in modulating migratory behaviour. Wind speed and direction affect both migratory behaviour (Afán et al., 2019) ~~as~~ AND seabird detectability by the observers [29]. Data on SST and wind were obtained through RNCEP package [54] in R [55] which allowed to request data from the NCEP/NCAR Reanalysis dataset (NOAA ESRL Physical Sciences Division; https://www.esrl.noaa.gov/psd/data/gridded/data.ncep.reanalysis2.html) for a specified range of space and time based on the observations of shearwaters. Specifically, we requested daily values at midday at the surface level, since Balearic shearwaters tend to migrate at very low height [29]. Variables requested were “air.995” (i.e. air temperature), “uwind.sig995” (i.e., u-wind component) and “vwind.995” (i.e. v-wind component). RNCEP interpolates the nearest data value (in terms of location and time) in the NCEP/NCAR dataset to provide the requested information. We also considered the standard deviation of the meteorological values (i.e. u-wind, v-wind and temperature) used for this interpolation as additional predictors in our models (Table 1). General flight activity of seabirds increases during moonlit nights and moon phase has been shown to affect shearwater migration behaviour [40]. The daily fraction of the moon illuminated at midnight was sourced by the U.S Naval Observatory & Astronomical Applications Department (<http://aa.usno.navy.mil/data/>).

Together with the usual interannual variability in food resources, long-term climate change may also affect~~s~~ the at-sea distribution of this species [43,47,56] likely through effects on fish stocks [57]. The possible impact of climate change in the distribution of shearwaters at a regional level was ~~took~~ TAKEN into account through the North Atlantic Oscillation Index (NAO; [58]) obtained from the Climate Prediction Center (US National Weather Service, NOAA), as monthly data from 1950 to 2018 (<http://www.cpc.ncep.noaa.gov/products/precip/CWlink/pna/nao.shtml>). Monthly values rather THAN daily NAO index was preferred in order to avoid correlation with SST.

Water depth has been shown to influence seabird distributions [59]. Bathymetry was used as a surrogate of coastal-pelagic areas (Afán et al., 2019). Bathymetric data were sourced by European Marine Observation and Data Network (EMODnet; <http://www.emodnet-bathymetry.eu/data-products>) at ~200 m resolution although they were later aggregated through a moving-average window to 10 km resolution in order to obtain a more general bathymetry pattern regarding the shearwater observation location.

In addition to the previous environmental predictors, we considered spatial coordinates (longitude and latitude), as well as date (i.e., julian date) and year of the observation as predictors modelling the seasonal and interannual variation in shearwater abundance, respectively.

**Statistical analysis**

To avoid collinearity, from the total set of environmental predictors only uncorrelated variables (i.e., non-significant Pearson correlation coefficient between pairs of variables) were kept in the models.

We applied machine-learning techniques to predict shearwater abundance, specifically Random Forest models. This choice was based on a previous assessment of eight different modelling techniques carried out on the same dataset [60]. We used packages caret [61] and randomForest [62] in R [55] to build the models. We built separated models for pre-breeding and post-breeding migration. As usual in count data, abundance of shearwaters followed a Poisson distribution [63]. Therefore, prior to build our models we log-transformed the abundance data in order to improve the accuracy of our results.

Random forest models apply bagging (i.e., bootstrap aggregating) to sub-sample the data that are used for training. We assessed the performance of the models based on the accuracy of predictions for the independent test data derived from the Out- of-bag (OOB) error. [64]. To determine the parameter values offering the best fit, we specified a set of tuning values to be tested in the training subset during the calibration of the models. RMSE was used to select the optimal parameter value. We also used RMSE as a measure of the accuracy of each particular model. As an additional assessment of the models, we quantified the amount of variance explained (R^2^). To evaluate how well the models were able to predict the test set outcomes, we randomly split the abundance data into training and test data subsets by setting aside 20% of the data for testing the models [65]. The models were calibrated on the training data and then evaluated on the test data. Specifically, we quantified to what extent the abundance in test data agreed with the model predicted abundance by means of the Pearson correlation coefficient.

Relative variable importance of the variables in predicting shearwater abundance was assessed by means of the number of trees where the variable was included and the minimal depth of the variable in the tree (package randomForestExplainer in R; [66].

As an example of the potential marine areas that can be identified from the models, we predicted shearwater abundance across the study area on the fifteenth of the month, from May to December, based on THE environmental conditions DURING 2017.

**Results**

An exploratory analysis of the data showed that observations of Balearic shearwaters contained in the databases preceding the year 2005 and collected during 2018 were insufficient to model daily patterns during the migration periods. After the removal of data recorded before 2005 and after 2017, 7,492 and 4,510 observations remained for the pre-breeding and post-breeding migration periods, respectively. These observations covered the entire non-breeding range of the species (Fig 1).

After inspection of collinearity (i.e., significant Pearson correlation coefficient between pairs of variables), we did not find highly correlated variables (r<0.44 for the total set of environmental predictors in Table 1), thus all the predictors considered in the initial set were kept for model selection.

According to the results derived from the bagging procedure, there was substantial variation (measured as 95% confidence interval) among models built with different training data subsamples, particularly during the post-breeding migration (Fig 2). Accuracy (RMSE) of the pre-breeding model was 3% larger compared with the post-breeding model (Bonferroni’s p-value adjustment < 0.01). Contrastingly, differences in the variance explained (R^2^) between the pre-breeding and the post-breeding migration models were not statistically significant (Bonferroni’s p-value adjustment = 0.7314). Predictive ability was substantially high during the post-breeding period, when the correlation between observed and predicted abundance was 0.71, whereas the correspondence between observed and predicted values was 0.68 in the pre-breeding model. Models built with the total dataset for both pre-breeding and post-breeding migration explained around 53% of the variation in shearwater abundance (computed from bootstrap resampling with 25 repetitions).

**Fig. 2. Model comparison.** Comparison of resamples (bagging) between pre- (RF pre) and post-breeding (RF post) models. *RMSE*: Root Mean Squared Error; *Rsquared*: % variance explained. Mean and Confidence Interval (95%).

Variable importance differed between migration periods (Fig 3), with a larger importance of chlorophyll concentration during the post-breeding migration. Bathymetry showed a moderate contribution to the abundance of shearwaters during both migration movements. Interannual variability (i.e., effect of “year”) in the spatial abundance pattern was also relevant during pre-breeding and post-breeding migration. In relation to weather predictors, temperature and wind were variables significantly affecting the abundance of shearwaters during both migration periods. In contrast, lunar cycle and NAO index hardly contributed to the abundance patterns described in the models.

**Fig. 3. Relative importance of the variables.** Relative variable importance (in terms of number of trees and minimum depth) in the random forest models predicting Balearic shearwater abundance. a) pre-breeding migration; b) post-breeding migration. *times_a_root*: total number of trees in which the variable is used for splitting the root node. *mean_minimal_depth*: mean minimal depth. Ten top variables are highlighted in blue. The size of points reflects the number of nodes split on the variable.

According to the predicted abundance of shearwaters for different values of year and latitude, the number of migrating shearwaters appeared to be larger at higher latitudes in recent years, although there was no clear trend over the study period (Fig 4).

**Fig. 4.** **Interactions between variables.** Predicted abundance of shearwaters for different values of year and latitude. (a) pre-breeding; (b) post-breeding migration models.

Spatial predictions derived from pre- and post-breeding models (Fig 5) showed how abundance of shearwaters decreases at sea locations in the Mediterranean from May to August and then it gradually increases again from September to December. However, according to the models, Balearic Shearwaters use slightly different regions during their northward (May–August) and southward migration (September–December). Our models supported that Alboran Sea is a key area for migrating shearwaters, particularly during post-breeding migration. Similarly, Gulf of Cadiz also showed high concentrations of shearwaters, specially during the pre-breeding movement. Our results suggested that Atlantic coast of Portugal and France are migratory stopover and/or moulting sites for Balearic shearwaters both during pre and post-breeding migration. The post-breeding model showed that Western English Channel is an important area for Balearic shearwaters mainly during August, whereas high abundance of shearwaters is predicted along the Algerian/Tunisian coastline in November.

**Fig. 5.** **Spatial predictions of shearwater distribution during migration.** Probability of shearwater abundance (calculated as a percentage based on maximum predicted abundance) across the study area on the fifteenth of the month, from May to December, from environmental conditions (see Table 1) occurred in year 2017.

**Discussion**

Here we undertook the first long-term study on the spatio-temporal patterns of migrating Balearic shearwaters from data gathered within the framework of citizen science projects. Our results showed that data obtained in initiatives such as Trektellen and eBird, together with large-scale open datasets, can be successfully applied to describe the migratory distribution of seabird species accurately. Previous research found Balearic shearwater abundance to be extremely difficult to predict and it failed to provide reliable predictions on the spatial distribution of shearwater numbers [10]. In contrast to this earlier modelling attempt based on ship transect data, our models provided strongly correlated predictions with the observed abundances and explained a significant proportion of the variance existing in the numbers of migrating shearwaters.

In any research, the value of the derived knowledge directly relies on the quality of the data used. Direct observations of birds collected by volunteers are a cost-effective source of data that have been previously applied to develop spatial distribution maps of seabirds [67]. Nevertheless, observations of migratory movements collected from coastal land-based observatories have been claimed to be fragmentary and biased thus they have been frequently considered to be deficient in determining migratory patterns [29]. In recent years, however, the new Big Data approach has led to an increase interest in gathering massive quantities of datasets frequently shared over the Internet. As other Big Data, observations derived from citizen initiatives frequently exhibit noise affecting their quality [68]. To deal with such a large amount of data and its characteristic noise, machine learning techniques (i.e., the application of computational methods underlying experience-based decision making; [69], as those applied in our study, offer an efficient tool, bringing new opportunities to take advantage of these massive data [70]. In contrast to alternative approaches such as the use of tracking data [19,20,71] or vessel surveys [9,10], these massive datasets provide with low-cost information on long-term temporal and large spatial extents which allow to identify spatio-temporal patterns that are representative of many different individuals belonging to several populations (e.g., [26] as well as to quantify their seasonal and interannual variation in the long-term.

Supporting the validity of our models results, the spatial predictions obtained are consistent with previous findings on important marine areas for shearwaters identified from tracking data and vessel surveys [9,10,20], even if our predictions should be taken rather as an illustration than as an exact calculation, since they are based on a single snapshot on the fifteenth of the month in a particular year. However, the major achievement of our modelling attempt is the high temporal resolution that we achieved with our models and the possibility of deriving spatial predictions that take into consideration the spatio-temporal heterogeneity in migration patterns for specific time frames (even for future projections). In this sense, most of the studies predicting animal spatial patterns have been focused on the stationary distribution in more or less temporally invariable environments (but see [72,73]. In contrast, marine environment is highly dynamic and its conditions in a particular location are ever changing [74]. New available global data from satellite imagery are frequently provided at high spatial and temporal resolutions, offering an excellent opportunity to model highly mobile migratory species by informing on the heterogeneity at different spatio-temporal scales affecting the en-route habitat use [23]. This high-resolution information allows modelling daily or even hourly conditions at specific locations, such as in our study. We show that these data, together with observations of birds collected in citizen science projects, can be used to identify conservation concerns and targets at regional levels related to environmental changes such as global warming or fisheries (Martín et al., 2014; Paleczny et al., 2015), among others, that are difficult to track using traditional approaches.

**Differences between pre- and post-breeding migration**

In addition, we show that predictive models based on volunteer data not only provide accurate predictions but they contribute to a better understanding of the factors modulating the migratory movements and to identify the seasonal and interannual variability existing in the at sea locations during migration. Among others, fluctuations in migration are usually related to changes in food resources [47]. Differences in chlorophyll-a concentration and bathymetry of the at sea distribution of Balearic shearwaters between the two migration movements suggest that birds leaving the Mediterranean target shallower waters where productivity is higher, whereas shearwaters returning to the breeding grounds appear to be less conditioned on food availability. Most likely, this is because, after breeding, migrating birds need to replenish energy reserves, thus they will select key stopover locations along the trip where maximize their refueling opportunities [46]. This stronger link between shearwater abundance and food availability likely lead to the larger variability observed in the spatio-temporal patterns of shearwaters during post-breeding compared to the pre-breeding migration.

Spatial long-term trends in abundance related to changes in fisheries and/or in climate affecting the Balearic shearwater distribution over the study period can be inspected from the predicted abundance of shearwaters for different values of year and latitude. Although there is no a straightforward pattern over the study period, it seems that the abundance of the Balearic shearwater has increased in northern latitudes in recent years. These results are consistent with the northward shift in the spatial range of Balearic shearwaters along northwest European coasts that has been found in previous studies [43,47,56], apparently not directly related to climate change but meditated by recent changes in prey fish and discard availability [9].

**Predictive models for delimiting marine protected areas**

Understanding the spatial distribution of seabirds at sea is crucial for the protection of these species and their habitats [1]. However, whereas the terrestrial breeding areas where seabirds occur are usually well known, a gap still exists in the identification of protected areas at sea (e.g., Natura 2000 network; [76]. Identification of marine protected areas for seabirds and other highly mobile marine species is a difficult task [77] because their movements at sea and the spatio-temporal variability associated remain largely unknown [71]. In this sense, a number of more or less successful approaches have been developed to identify marine Important Bird and biodiversity Areas (IBAs), which are important foraging, migrating or wintering sites for seabirds [78]. Our model predictions can be useful for delimiting important areas for the conservation of the Balearic shearwater. Specifically, mapping model average and/or accumulate predictions over seasons and/or particular interannual time frames can provide useful information on migratory routes and stopover sites, as well as on the seasonal and interannual variability in such factors and patterns. In addition, from predictions based on upcoming environmental conditions at sea, we can assess how climate and other human-induced changes may affect the future habitat use made by shearwaters.

**Caveats regarding data availability and predictive ability**

According to our results, the predictive ability of the models describing Balearic shearwater abundance patterns (particularly during post- breeding migration, when variability in abundance is larger) is subjected to the dataset used in the model calibration. Balearic shearwater is a rare but a charismatic species which generates high interest among birdwatchers. In addition, its mostly coastal spatial distribution facilitates an abundant record of this species from land-based sites throughout its annual cycle. In contrast, models built for other species less well-known and/or more difficult to observe could not offer as good results as those here shown for Balearic shearwaters. However, according to our results and considering the increasing amount of data collected by volunteers in the framework of citizen science initiatives all over the world, these datasets should be taken into consideration when studying highly mobile animals.

**Conclusions**

We show that data gathered in citizen science initiatives together with recently available high-resolution satellite imagery may offer a powerful and cost-effective tool for the long-term spatial monitoring of the migratory patterns in sensitive marine species. Due to cost and logistic constraints, long-term monitoring of seabirds is scarce [79]. In addition, seabird behavior at sea is complex, with high variability in the locations mainly driven by spatial and temporal changes in food availability affecting the size and the shape of the areas used. As an alternative to more traditional approaches [78], modelling techniques can provide a comprehensive picture, both spatially and temporally, of the migratory patterns at the population level that can be complementary to the detailed information at individual level obtained from tracking data. Similar machine learning techniques as those applied in this study, may contribute to extract information from other existing and future datasets collected by volunteers and inform marine spatial planning at regional spatial scales for multiple species. Modelling approaches can also become key tools to detect the impacts of climate and other global changes in the at sea distribution of this and other marine species. In this sense, other seabird and sensitive vertebrate marine taxa of conservation concern such as sea turtles [80] and marine mammals [81] could also benefit from our approach.

**Aknowledgements**

This study is part of a research project (“Environmental factors determining the interannual variation in the migration of Balearic and Scopoli’s shearwaters in the Mediterranean”, 2018-2019) which form part of the Annual Programme of Grants of the *Instituto de Estudios Ceutíes* (IEC, Autonomous City of Ceuta, Spain), years 2018-2019.

**References**

1.	Zacharias MA, Roff JC. Use of focal species in marine conservation and management: a review and critique. Aquatic Conservation: Marine and Freshwater Ecosystems. 2001;11: 59–76. doi:10.1002/aqc.429

2.	Lascelles B, Rice J, Sato M, Tarzia M, Wanless R. The First Global Integrated Marine Assessment - World Ocean Assessment I - Chapter 38. Seabirds. 2016.

3.	Daunt F, Mitchell I. Impacts of climate change on seabirds. MCCIP Science Review. 2013; 125–133. doi:doi:10.14465/2013.arc14.125-133

4.	Burger A, Shaffer S. Application of Tracking and Data-Logging Technology in Research and Conservation of Seabirds. The Auk. 2008;125: 253–264.

5.	Rodríguez A, Arcos JM, Bretagnolle V, Dias MP, Holmes ND, Louzao M, et al. Future Directions in Conservation Research on Petrels and Shearwaters. Frontiers in Marine Science. 2019;6: 94. doi:10.3389/fmars.2019.00094

6.	Hutto RL. On the Importance of Stopover Sites to Migrating Birds. The Auk. 1998;115: 823–825. doi:10.2307/4089500

7.	Halupka L, Wierucka K, Sztwiertnia H, Klimczuk E. Conditions at autumn stopover sites affect survival of a migratory passerine. Journal of Ornithology. 2017;158: 979–988. doi:10.1007/s10336-017-1472-5

8.	Arroyo GM, Mateos-Rodríguez M, Muñoz AR, De la Cruz A, Cuenca D, Onrubia A. New population estimates of a critically endangered species , the Balearic Shearwater Puffinus mauretanicus , based on coastal migration counts. Bird Conservation International. 2014;26: 87–99. doi:10.1017/S095927091400032X

9.	Jones A, Wynn RB, Yésou P, Thébault L, Collins P, Suberg L, et al. Using integrated land- and boat-based surveys to inform conservation of the Critically Endangered Balearic shearwater Puffinus mauretanicus in northeast Atlantic waters. Endangered Species Research. 2014;25.

10.	Oppel S, Meirinho A, Ramírez I, Gardner B, O’Connell AF, Miller PI, et al. Comparison of five modelling techniques to predict the spatial distribution and abundance of seabirds. Biological Conservation. 2012;156: 94–104. doi:10.1016/j.biocon.2011.11.013

11.	Walker RH, Robinson RA, Leech DI, Moss D, Kew AJ, Barber LJ, et al. Bird ringing and nest recording in Britain and Ireland in 2013. Ringing & Migration. 2014;29: 90–150. doi:10.1080/03078698.2014.995529

12.	Guilford T, Meade J, Willis J, Phillips RA, Boyle D, Roberts S, et al. Migration and stopover in a small pelagic seabird, the Manx shearwater Puffinus puffinus: insights from machine learning. Proceedings Biological sciences. 2009;276: 1215–1223. doi:10.1098/rspb.2008.1577

13.	Hedd A, Pollet IL, Mauck RA, Burke CM, Mallory ML, McFarlane Tranquilla LA, et al. Foraging areas, offshore habitat use, and colony overlap by incubating Leach’s storm-petrels Oceanodroma leucorhoa in the Northwest Atlantic. PLOS ONE. 2018;13: e0194389. doi:10.1371/journal.pone.0194389

14.	Ramos R, Carlile N, Madeiros J, Ramírez I, Paiva VH, Dinis HA, et al. It is the time for oceanic seabirds: Tracking year-round distribution of gadfly petrels across the Atlantic Ocean. Diversity and Distributions. 2017;23: 794–805. doi:10.1111/ddi.12569

15.	Fiedler W, Radolfzell V. New technologies for monitoring bird migration and behaviour. British Trust for Ornithology Ringing & Migration. 2009;24: 175–179. doi:10.1080/03078698.2009.9674389

16.	Weimerskirch H, Delord K, Guitteaud A, Phillips RA, Pinet P. Extreme variation in migration strategies between and within wandering albatross populations during their sabbatical year, and their fitness consequences. Scientific Reports. 2015;5: 8853.

17.	Martín B, Onrubia A, Ferrer M. Effects of climate change on the migratory behavior of the common buzzard Buteo buteo. Clim Res. 2014;60: 187–197.

18.	Scholer MN, Martín B, Ferrer M, Onrubia A, Bechard MJ, Kaltenecker GS, et al. Variable shifts in the autumn migration phenology of soaring birds in southern Spain. Ardea. 2016; in press-in press. doi:10.5253/arde.v104i1.a

19.	Afán I, Navarro J, Grémillet D, Coll M, Forero M. Maiden voyage into death: are fisheries affecting seabird juvenile survival during the first days at-sea? Royal Society Open Science. 31 Jan 20196: 181151. doi:10.1098/rsos.181151

20.	Guilford T, Wynn R, McMinn M, Rodríguez A, Fayet A, Maurice L, et al. Geolocators reveal migration and pre-breeding behaviour of the critically endangered balearic shearwater Puffinus mauretanicus. PLoS ONE. 2012;7: 1–8. doi:10.1371/journal.pone.0033753

21.	Pérez-Roda A, Delord K, Boué A, Arcos JM, García D, Micol T, et al. Identifying Important Atlantic Areas for the conservation of Balearic shearwaters: Spatial overlap with conservation areas. Deep Sea Research Part II: Topical Studies in Oceanography. 2017;141: 285–293. doi:10.1016/j.dsr2.2016.11.011

22.	Strasser B, Baudry J, Mahr D, Sanchez G, Tancoigne E. “Citizen Science”? Rethinking Science and Public Participation. Science & Technology Studies. 2019;32: 52–76.

23.	Pocock M, Chandler M, Bonney R, Thornhill I, Albin A, August T, et al. A Vision for Global Biodiversity Monitoring With Citizen Science. Advances in Ecological Research. 2018. doi:10.1016/bs.aecr.2018.06.003

24.	Chandler M, See L, Copas K, Bonde AMZ, López BC, Danielsen F, et al. Contribution of citizen science towards international biodiversity monitoring. Biological Conservation. 2017;213: 280–294. doi:10.1016/j.biocon.2016.09.004

25.	Kelling S, Gerbracht J, Fink D, Lagoze C, Wong W, Yu J, et al. A Human/Computer Learning Network to Improve Biodiversity Conservation and Research. AI Magazine. 2013;34: 10–20.

26.	Gouraguine A, Moranta J, Ruiz-Frau A, Hinz H, Reñones O, Ferse SCA, et al. Citizen science in data and resource-limited areas: A tool to detect long-term ecosystem changes. PLOS ONE. 2019;14: e0210007. doi:10.1371/journal.pone.0210007

27.	Kelling S, Johnston A, Fink D, Ruiz-Gutierrez V, Bonney R, Bonn A, et al. Finding the signal in the Noise of Citizen Science Observations. bioRxiv. 2018; 326314. doi:10.1101/326314

28.	BirdLife International. Puffinus mauretanicus. The IUCN Red List of Threatened Species 2018: e.T22728432A132658315. http://dx.doi.org/10.2305/IUCN.UK.2018-2.RLTS.T22728432A132658315.en. Downloaded on 15 March 2019; 2018.

29.	Martín B, Onrubia A, Ferrer M. Endemic shearwaters are increasing in the Mediterranean in relation to factors that are closely related to human activities. Global Ecology and Conservation. 2019;in press.

30.	Guisan A, C Edwards T, Hastie T. Generalized Linear and Generalized Additive Models in Studies of Species Distributions: Setting the Scene. Ecological Modelling. 2002;157: 89–100. doi:10.1016/S0304-3800(02)00204-1

31.	Troost G. Trektellen. 2017.

32.	Reyes-González J, Zaiková Z, Morera V, de Felipe F, Militão T, Dell’Ariccia G, et al. Migración y ecología espacial de las poblaciones españolas de pardela cenicienta. Monografía n^o^ 3 del programa Migra. Madrid: SEO/BirdLife; 2017.

33.	Le Mao P, Yésou P. The annual cycle of Balearic Shearwaters and western-Mediterranean Yellow-legged Gulls: some ecological considerations. In: Aguilar JS, Monbailliu X, Paterson A, editors. Status and conservation of seabirds Proceedings of the 2nd Mediterranean Seabird Symposium. 1993. pp. 135–145.

34.	Arcos J, Oro D. Significance of fisheries discards for a threatened Mediterranean seabird, the Balearic shearwater Puffinus mauretanicus. Mar Ecol Prog Ser. 2002;239: 209–220.

35.	Louzao M, García D, Rodríguez B, Abelló P. Evidence of krill in diet of Balearic Shearwaters Puffinus mauretanicus. Marine Ornithology. 2015;43: 49–51.

36.	eBird. eBird Basid Dataset. Version: EB_relNov-2017. Ithaca, New York: Cornell Lab of Ornithology; 2017.

37.	Mourino J, Arcos F, Salvadores R, Sandoval A, Vidal C. Status of the Balearic shearwater (Puffinus mauretanicus) on the Galician coast (NW Iberian Peninsula). Scientia Marina. 2003;67: 135–142.

38.	Yésou P. Recent changes in the summer distribution of the Balearic shearwater Puffinus mauretanicus off western France. 2003;67: 143–148.

39.	Afán I, Navarro J, Cardador L, Ramirez F, Kato A, Rodríguez B, et al. Foraging movements and habitat niche of two closely related seabirds breeding in sympatry. Marine Biology. 2014;161: 657–668. doi:10.1007/s00227-013-2368-4

40.	Dias MP, Granadeiro JP, Catry P. Do Seabirds Differ from Other Migrants in Their Travel Arrangements? On Route Strategies of Cory’s Shearwater during Its Trans-Equatorial Journey. PLOS ONE. 2012;7: e49376. doi:10.1371/journal.pone.0049376

41.	Freeman Robin, Dean Ben, Kirk Holly, Leonard Kerry, Phillips Richard A., Perrins Chris M., et al. Predictive ethoinformatics reveals the complex migratory behaviour of a pelagic seabird, the Manx Shearwater. Journal of The Royal Society Interface. 2013;10: 20130279. doi:10.1098/rsif.2013.0279

42.	Louzao M, Igual JM, McMinn M, Aguilar JS, Triay R, Oro D. Small pelagic fish, trawling discards and breeding performance of the critically endangered Balearic shearwater: Marine Ecology Progress Series. 2006;318: 247–254.

43.	Luczak C, Beaugrand G, Jaffré M, Lenoir S. Climate change impact on Balearic shearwater through a trophic cascade. Biology Letters. 2011;7: 702–705. doi:10.1098/rsbl.2011.0225

44.	Ramos R, Granadeiro JP, Nevoux M, Mougin J-L, Dias MP, Catry P. Combined Spatio-Temporal Impacts of Climate and Longline Fisheries on the Survival of a Trans-Equatorial Marine Migrant. PLOS ONE. 2012;7: e40822. doi:10.1371/journal.pone.0040822

45.	Robinson J, Dornelas M, Ojanguren AF. Interspecific synchrony of seabird population growth rate and breeding success. Ecology and evolution. 2013;3: 2013–9. doi:10.1002/ece3.592

46.	McKnight A, Allyn A, Duffy D, Irons D. ‘Stepping stone’ pattern in Pacific Arctic tern migration reveals the importance of upwelling areas. Mar Ecol Prog Ser. 2013;491: 253–264.

47.	Wynn RB, Josey S, Martin A, Johns D, Yésou P. Climate-driven range expansion of a critically endangered top predator in northeast Atlantic waters. Biology Letters. 2007;3: 529–532. doi:10.1098/rsbl.2007.0162

48.	Benoit-Bird KJ, Battaile BC, Heppell SA, Hoover B, Irons D, Jones N, et al. Prey Patch Patterns Predict Habitat Use by Top Marine Predators with Diverse Foraging Strategies. PLOS ONE. 2013;8: e53348. doi:10.1371/journal.pone.0053348

49.	Frederiksen M, Edwards M, Richardson AJ, Halliday NC, Wanless S. From plankton to top predators: bottom-up control of a marine food web across four trophic levels. Journal of Animal Ecology. 2006;75: 1259–1268. doi:10.1111/j.1365-2656.2006.01148.x

50.	Gons HJ, Auer MT, Effler SW. MERIS satellite chlorophyll mapping of oligotrophic and eutrophic waters in the Laurentian Great Lakes. Remote Sensing of Environment. 2008;112: 4098–4106. doi:10.1016/j.rse.2007.06.029

51.	Cortés V, García-Barcelona S, González-Solís J. Sex- and age-biased mortality of three shearwater species in longline fisheries of the Mediterranean. Marine Ecological Progress Series. 2018;588: 229–241.

52.	Genovart M, Bécares J, Igual J-M, Martínez-Abraín A, Escandell R, Sánchez A, et al. Differential adult survival at close seabird colonies: The importance of spatial foraging segregation and bycatch risk during the breeding season. Global Change Biology. 2018;24: 1279–1290. doi:10.1111/gcb.13997

53.	Natale F, Gibin M, Alessandrini A, Vespe M, Paulrud A. Mapping Fishing Effort through AIS Data. PLOS ONE. 2015;10: e0130746. doi:10.1371/journal.pone.0130746

54.	Kemp MU, Emiel van Loon E, Shamoun-Baranes J, Bouten W. RNCEP: global weather and climate data at your fingertips. Methods in Ecology and Evolution. 2012;3: 65–70. doi:10.1111/j.2041-210X.2011.00138.x

55.	R Development Core Team. R: A language and environment for statistical computing. R Foundation for Statistical Computing. 2018.

56.	Votier SC, Bearhop S, Attrill MJ, Oro D. Is climate change the most likely driver of range expansion for a critically endangered top predator in northeast Atlantic waters? Biology Letters. 2008;4: 204–205. doi:10.1098/rsbl.2007.0558

57.	Tsikliras AC, Licandro P, Pardalou A, McQuinn IH, Gröger JP, Alheit J. Synchronization of Mediterranean pelagic fish populations with the North Atlantic climate variability. Deep Sea Research Part II: Topical Studies in Oceanography. 2019;159: 143–151. doi:10.1016/j.dsr2.2018.07.005

58.	Visbeck MH, Hurrell JW, Polvani L, Cullen HM. The North Atlantic Oscillation: Past, present, and future. Proc Natl Acad Sci USA. 2001;98: 12876. doi:10.1073/pnas.231391598

59.	Yen PPW, Sydeman WJ, Hyrenbach KD. Marine bird and cetacean associations with bathymetric habitats and shallow-water topographies: implications for trophic transfer and conservation. Journal of Marine Systems. 2004;50: 79–99. doi:10.1016/j.jmarsys.2003.09.015

60.	Martín B, González-Arias J, Vicente-Vírseda JA. Big Data for studying migration in endangered species. Empiria. under review.

61.	Kuhn M. Building Predictive Models in R Using the caret Package. Journal of Statistical Software. 2008;28: 1–26. doi:doi:http://dx.doi.org/10.18637/jss.v028.i05

62.	Liaw A, Wiener M. Classification and Regression by randomForest. R news. 2002;2: 18–22.

63.	Hilbe JM. Modeling Count Data. Cambridge: Cambridge University Press; 2014. doi:10.1017/CBO9781139236065

64.	Gareth J, Witten D, Hastie T, Tibshirani R. An Introduction to Statistical Learning. Springer; 2013.

65.	Araújo MB, Thuiller W, Pearson RG. Climate warming and the decline of amphibians and reptiles in Europe. Journal of Biogeography. 2006;33: 1712–1728. doi:10.1111/j.1365-2699.2006.01482.x

66.	Ishwaran H, Kogalur U, Gorodeski E, Minn A, Lauer M. randomForestExplainer: Explaining and Visualizing Random Forests in Terms of Variable Importance. 2010.

67.	Ward EJ, Marshall KN, Ross T, Sedgley A, Hass T, Pearson SF, et al. Using citizen-science data to identify local hotspots of seabird occurrence. Gandini P, editor. PeerJ. 2015;3: e704. doi:10.7717/peerj.704

68.	García-Gil D, Luengo J, García S, Herrera F. Enabling Smart Data: Noise filtering in Big Data classification. Information Sciences. 2019;479: 135–152. doi:10.1016/j.ins.2018.12.002

69.	Schain M. Machine Learning Algorithms and Robustness. Universitat Tel-Aviv; 2015. Available: https://books.google.es/books?id=dWTRnQAACAAJ

70.	Martínez–Álvarez F, Morales–Esteban A. Big data and natural disasters: New approaches for spatial and temporal massive data analysis. Computers & Geosciences. 2019;129: 38–39. doi:10.1016/j.cageo.2019.04.012

71.	Meier RE, Wynn RB, Votier SC, McMinn Grivé M, Rodríguez A, Maurice L, et al. Consistent foraging areas and commuting corridors of the critically endangered Balearic shearwater Puffinus mauretanicus in the northwestern Mediterranean. Biological Conservation. 2015;190: 87–97. doi:10.1016/j.biocon.2015.05.012

72.	Martín B, Alonso JC, Martín CA, Palacín C, Magaña M, Alonso JA. Influence of spatial heterogeneity and temporal variability in habitat selection : A case study on a great bustard metapopulation. Ecological Modelling. 2012;228: 39–48. doi:10.1016/j.ecolmodel.2011.12.024

73.	Zurell D, Jeltsch F, F. Dormann C, Schröder B. Static species distribution models in dynamically changing systems: How good can predictions really be? Ecography. 2009;32: 733–744. doi:10.1111/j.1600-0587.2009.05810.x

74.	Zipkin E, Gardner B, Gilbert A, O’Connell A, Royle J andrew, D Silverman E. Distribution patterns of wintering sea ducks in relation to the North Atlantic Oscillation and local environmental characteristics. Oecologia. 2010;163: 893–902. doi:10.1007/s00442-010-1622-4

75.	Paleczny M, Hammill E, Karpouzi V, Pauly D. Population Trend of the World’s Monitored Seabirds, 1950-2010. PLOS ONE. 2015;10: e0129342. doi:10.1371/journal.pone.0129342

76.	European Commission. Report from the Comission to the European Parliament and the Council on the progress in establishing marine protected areas (as required by Article 21 of the Marine Strategy Framework Directive 2008/56/EC). 2015.

77.	Ronconi R, Lascelles B, M. Longham G, B. Reid J, Oro D. The role of seabirds in Marine Protected Area identification, delineation, and monitoring: Introduction and synthesis. Biological Conservation. 2012;156: 1–4. doi:10.1016/j.biocon.2012.02.016

78.	Soanes LM, Bright JA, Angel LP, Arnould JPY, Bolton M, Berlincourt M, et al. Defining marine important bird areas: Testing the foraging radius approach. Biological Conservation. 2016;196: 69–79.

79.	Becker P, Chapdelaine G. Further development of seabird monitoring. In: Tasker M, Furness R, editors. Seabirds Monitors of the Marine Environment ICES Cooperative Research Report. Copenhagen, Denmark: International Council for the Exploration of the Sea No. 258; 2003. pp. 52–60.

80.	Casale P, C. Broderick A, Antonio Camiñas J, Cardona L, Carreras C, Demetropoulos A, et al. Mediterranean sea turtles: current knowledge and priorities for conservation and research. Endangered Species Research. 2018;36: 229–267.

81.	Gonzalvo J, Valls M, Cardona L, Aguilar A. Factors determining the interactions between bottom trawlers off the Balearic Archipelago (western Mediterranean Sea). Journal of Experimental Marine Biology and Ecology. 2008;36: 47–52. doi:10.1016/j.jembe.2008.08.013
