# Supplementary material for: Citizen science for predicting spatio-temporal patterns in seabird abundance during migration
Source: PLoS One. 2020 Aug 14;15(8):e0236631. doi: 10.1371/journal.pone.0236631 (PMC7428152; doi:10.1371/journal.pone.0236631)
Supplement: Attachment — Submitted filename: answer_to_reviewers_PONE_Feb2020.docx [file pone.0236631.s003.docx]

22^nd^ July 2019

PONE-D-19-29580
Citizen science and machine learning for predicting spatio-temporal patterns in seabird migration

David Hyrenbach, Ph.D.
Academic Editor
PLOS ONE

PLOS ONE

Dear Dr. Hyrenbach,

We sincerely thank you for allowing us to resubmit our paper. Following your instructions, we resubmit the manuscript titled “Citizen science and machine learning for predicting spatio-temporal patterns in seabird migration” (PONE-D-19-29580), now retitled “Citizen science for predicting spatio-temporal patterns in seabird abundance during migration”, after fully addressing all the concerns raised by you and the reviewer.

Changes done throughout the text have been recorded, via track-changes, in a document enclosed with this re-submission. Please find below the detailed explanation of the modifications we have made in response to the concerns raised. A copy of the document with the *track changes* feature turned off is also attached. Lines indicated below and within the document with the *track changes feature* turned on, referred to the document with the *track changes* feature turned off.

We hope that you will find this revised version suitable for publication in *PLOS ONE*.

Yours faithfully,

Beatriz Martín on behalf of all the authors

PONE-D-19-29580
Citizen science and machine learning for predicting spatio-temporal patterns in seabird migration
PLOS ONE

Dear Dr Martin,

Thank you for submitting your manuscript to PLOS ONE. After careful consideration, we feel that it has merit but does not fully meet PLOS ONE’s publication criteria as it currently stands. Therefore, we invite you to submit a revised version of the manuscript that addresses the points raised during the review process.

i apologize for the long handling time, but securing reviewers proved very difficult.  Because I was only able to secure one review, I did another review of the ms, and I concluded that it needed substantial revisions before it could be published.  I forward an annotated version with inserted comments, edits, and questions.  In particular, I urge the authors to:

1) revise the introduction to remove redundancies / and focus focus on the main goals.

**Authors**: Done. See changes made throughout the Introduction section.

2) revise the introduction and discussion to ensure the limitations of citizen science data are acknowledged\. currently, the ms highlights the limitations of tracking and vessel-based surveys, but there are many similar limitations to citizen science data: uncertainty of identification, uncertainty of counts, uneven coverage and lack of systematic effort, and lack of knowledge of the colony provenance of the birds sighted at sea.

**Authors**: Done. See changes made in the Introduction and Discussion sections.

3) revise the methods section (and augment with supplementary tables) to provide readers with a sense of how the sighting / fishery / environmental datasets were cleaned / processed. in particular, address the uncertainties associated with the species identifications, and explicitly explain how the fishery data were subsetted by sector / fishery / target.

**Authors**: New figures have been added both in Methods (see new Figure 1) and in a new Supplementary Material section (Figures S1, S2 and S3), showing the temporal and spatial representation of the data, as well as the count effort distribution over the days of the week (as requested by the reviewer#1, see answer to comments below regarding this issue). In addition, the results of the collinearity assessment are also now reported in the Supplementary Material. Balearic shearwater generates much attention both from amateur and expert ornithologists thus it is usually well-known species and correctly identified when sighted. However, we cannot totally exclude species identification errors among the different sources of variation associated with citizen science data. The previous is now stated in the text (see lines 167-170). Regarding the data on fisheries, JRC provides a detailed map of high intensity fisheries areas in 2014-2015 in Europe from tracking data of fishing vessels. The map allows to identify which are the areas where they fish more frequently. Vessel tracking data was derived from the open source Automatic Identification System (AIS), allowing to analyse the relation between fishing communities and fishing areas at high spatial resolution across Europe. Data used to build the map consist of 150 million positions from European fishing vessels above 15 m in length. This map was created to be used by scientist and experts for fisheries management and fisheries research both from an environmental and a socio-economic perspective. We have now clarified all that in the text.

4) furthermore, please provide evidence that the authors had cleared the terms of use of these publicly available sighting data, and are thus able to publish these datasets.  moreover, explicitly state how many records / birds were included in each dataset, for the pre and post breeding migration. finally, explicitly state whether "zero" counts were included, if standardized surveys (or sightings of other species were reported, but no Balearic Shearwatyer sightings were reported).

**Authors**: Terms of use of eBird database have been cleared (https://www.ala.org.au/wp-content/uploads/2015/02/Terms_of_Use.v3.pdf). Trektellen is a public database of migration / seawatch counts and ringing results (see additions into the Acknowledge section). On the other hand, only records attributed to Balearic shearwaters were analysed. Both systematic and opportunistic data were analysed. Although it was possible to differentiate opportunistic and systematic surveys within the dataset, we opted for keeping all records for our analysis in order to test the robustness of the modelling approach in case our methods will be extended to other species for which this information is not available. This has now clarified in the text (see lines 171-174). In addition, we modelled species abundance with only abundance-given-presence (Pearce & Boyce 2006), thus zeros were not included in the data matrix (see lines 192-193). On the other hand, after the removal of data recorded before 2005 and after 2017, 7,492 and 4,690 observations (i.e., rows in the data matrix) remained for the pre-breeding and post-breeding migration periods, respectively. Number of records (as well as number of birds) considered in the analysis after removal are reported in the Results section (see lines 266-272).

5) the methods also need to be strengthened, since the ms refers to several exploratory analyses (daily abundances used to censor the study period and co-linearity of explanatory variables), but no results are provided. I urge the authors to provide supplementary tables with these results, so the readers can evaluate what was done.

**Authors:** Please see changes made throughout the Methods section as well as the new Supplementary Material attached to the manuscript including new figures reporting these issues.

6) the results also need to be strengthened, to ensure tests are clearly reported and evaluated. in particular, please use the comments added to the ms to address the comparison of the model R-squared values, and the correlations or modeled / observed abundances. finally, please justify the small number of iterations used (25) and the use of means +-/ 95% CI for some of the estimated parameters, rather than medians (25% - 75%).

**Authors:** We have followed the recommendations made throughout the document and we have clarified the comparisons and the tests performed (please see answers to comments embedded in the manuscript with track changes on). Regarding iterations and 95% CI, as it is stated in the Methods section, any random forest model apply bagging (i.e., bootstrap aggregating) to sub-sample the data that are used for training, thus each new tree is fit from a bootstrap sample of the training observations zi=(xi,yi). Therefore, using 25 repetitions of this bootstrap resampling actually means a lot of different trees to be built. Large data samples tend to approximate the normal distribution thus we think that the use of 95% CI is correct.

7) The discussion and conclusions were clear and concise, but they are difficult to evaluate , given the paucity of details in the methods / results.  Finally, I urge the authors to carefully consider how they identify "key" areas, and to consider that they are only using counts; there are no behavioral data (eg., feeding versus transiting).  Namely, would a high flux area (e.g., Gibraltar Strait) with lower counts be more sensitive to impacts than a foraging area with higher counts but lower flux?

**Authors**: Details have added in Methods and Results section following the recommendations of both the editor and the reviewer. Regarding the key areas, although we are analysing no behavioural data, to some extent, we can infer feeding and transiting areas from variable interactions. For instance, locations with tailwinds, high abundance level and low chlorophyll concentration can be interpreted as locations with high flux of birds, in contrast to locations with moderate abundance, no winds or moderate winds and high chlorophyll concentration levels. We have added the previous information in the new version of the manuscript (see lines 388-392). In addition, for the sake of clarity, we have moved the content related to this issue contained at the end of the Results section into the Discussion section, because we actually were discussing our results in relation to previous findings (see lines 392-399).

We would appreciate receiving your revised manuscript by Feb 22 2020 11:59PM. When you are ready to submit your revision, log on to https://www.editorialmanager.com/pone/ and select the 'Submissions Needing Revision' folder to locate your manuscript file.

If you would like to make changes to your financial disclosure, please include your updated statement in your cover letter.

To enhance the reproducibility of your results, we recommend that if applicable you deposit your laboratory protocols in protocols.io, where a protocol can be assigned its own identifier (DOI) such that it can be cited independently in the future. For instructions see: <http://journals.plos.org/plosone/s/submission-guidelines#loc-laboratory-protocols>

Please include the following items when submitting your revised manuscript:

- A rebuttal letter that responds to each point raised by the academic editor and reviewer(s). This letter should be uploaded as separate file and labeled 'Response to Reviewers'.
- A marked-up copy of your manuscript that highlights changes made to the original version. This file should be uploaded as separate file and labeled 'Revised Manuscript with Track Changes'.
- An unmarked version of your revised paper without tracked changes. This file should be uploaded as separate file and labeled 'Manuscript'.

Please note while forming your response, if your article is accepted, you may have the opportunity to make the peer review history publicly available. The record will include editor decision letters (with reviews) and your responses to reviewer comments. If eligible, we will contact you to opt in or out.

We look forward to receiving your revised manuscript.

Kind regards,

David Hyrenbach, Ph.D.
Academic Editor
PLOS ONE

Journal Requirements:

When submitting your revision, we need you to address these additional requirements.

1. Please ensure that your manuscript meets PLOS ONE's style requirements, including those for file naming. The PLOS ONE style templates can be found at http://www.plosone.org/attachments/PLOSOne_formatting_sample_main_body.pdf and <http://www.plosone.org/attachments/PLOSOne_formatting_sample_title_authors_affiliations.pdf>

2. Thank you for stating in your Funding Statement:

'This study is part of a research project (“Environmental factors determining the interannual variation in the migration of Balearic and Scopoli’s shearwaters in the Mediterranean”, 2018-2019) which has been partly financed by the Annual Programme of Grants of the Instituto de Estudios Ceutíes (IEC, Autonomous City of Ceuta, Spain), years 2018-2019.'

a. Please provide an amended statement that declares *all* the funding or sources of support (whether external or internal to your organization) received during this study, as detailed online in our guide for authors at <http://journals.plos.org/plosone/s/submit-now>.  Please also include the statement “There was no additional external funding received for this study.” in your updated Funding Statement.

b. Please include your amended Funding Statement within your cover letter. We will change the online submission form on your behalf.

**Authors:** Done.

[Note: HTML markup is below. Please do not edit.]

Reviewers' comments:

Reviewer's Responses to Questions

**Comments to the Author**

1. Is the manuscript technically sound, and do the data support the conclusions?

The manuscript must describe a technically sound piece of scientific research with data that supports the conclusions. Experiments must have been conducted rigorously, with appropriate controls, replication, and sample sizes. The conclusions must be drawn appropriately based on the data presented.

Reviewer #1: Yes

2. Has the statistical analysis been performed appropriately and rigorously?

Reviewer #1: Yes

3. Have the authors made all data underlying the findings in their manuscript fully available?

The [PLOS Data policy](http://www.plosone.org/static/policies.action#sharing) requires authors to make all data underlying the findings described in their manuscript fully available without restriction, with rare exception (please refer to the Data Availability Statement in the manuscript PDF file). The data should be provided as part of the manuscript or its supporting information, or deposited to a public repository. For example, in addition to summary statistics, the data points behind means, medians and variance measures should be available. If there are restrictions on publicly sharing data—e.g. participant privacy or use of data from a third party—those must be specified.

Reviewer #1: Yes

4. Is the manuscript presented in an intelligible fashion and written in standard English?

PLOS ONE does not copyedit accepted manuscripts, so the language in submitted articles must be clear, correct, and unambiguous. Any typographical or grammatical errors should be corrected at revision, so please note any specific errors here.

Reviewer #1: Yes

5. Review Comments to the Author

Please use the space provided to explain your answers to the questions above. You may also include additional comments for the author, including concerns about dual publication, research ethics, or publication ethics. (Please upload your review as an attachment if it exceeds 20,000 characters)

**Reviewer #1:** In this manuscript, authors present a novel work to evaluate whether data from a long-term monitoring programme carried out by volunteers, i.e., a citizen science project, can be used to assess bird migratory patterns. They focused on an endangered seabird species endemic to the Mediterranean Sea that migrates to the Atlantic out of the breeding period. Authors relied on supervised machine learning technics to predict bird abundance, building up the models from data gathered at a network of bird census stations. Specifically, they used random forest regression models to evaluate the usefulness of citizen science data in predicting spatial and temporal abundance based on a set of predictors, including many satellite-derived features, reaching an accuracy of about 70%. Authors concluded that combining long-term citizen science with predictive modelling can be a reliable tool to assist long-term monitoring of sensitive species, to identify important areas and/or to detect trends.
The paper is well structured. The study is accurate and provides an interesting approach. As general comments, the major strength of this study relies on the source data used. Trektellen is an online database recording opportunistic seabird counts carried out by volunteers across Europe. Despite its potential, very few papers have made use of this long-term database. As a counterpart, there are also some weaknesses in the work that should be amended for publication. My main concern in this regard is that authors tend to overstate along the manuscript the importance of random forest (RF) to predict spatial and temporal abundance. RF is a really powerful algorithm (I personally really love it) based on an ensemble of decision trees, but its use is not new in the context of species distribution/abundance modelling (e.g. Oppel et al. (2012) Comparison of five modelling techniques to predict the spatial distribution and abundance of seabirds. Biological Conservation, 156, 94-104). In fact, recent approaches for species distribution modelling tend to build up a final output based on an ensemble of several algorithms (a.k.a. Ensemble Ecological Niche Models), including RF among them. See the “ssdm” package in R for details (Schmitt et al. (2017) ssdm: An r package to predict distribution of species richness and composition based on stacked species distribution models. Methods in Ecology and Evolution, 8(12), 1795-1803; and also, e.g., Pereira et al. (2018) Using a multi-model ensemble forecasting approach to identify key marine protected areas for seabirds in the Portuguese coast. Ocean & Coastal Management, 153, 98-107.).

**Authors**: We thanks the reviewer for the mentioned publications in relation to the subject of our study. In fact, Oppel et al. (2012) was cited and discussed in our manuscript (see lines 108, 345-351). However, this previous research found Balearic shearwater abundance to be extremely difficult to predict and it failed to provide reliable predictions on the spatial distribution of shearwater numbers. Moreover, their predictions were based on abundance and absence data. In contrast to this earlier modelling attempt based on ship transect data, our models, based on abundance given presence, provided strongly correlated predictions with the observed abundances and explained a significant proportion of the variance existing in the numbers of migrating shearwaters.

In relation to the other studies mentioned by the reviewer (Schmitt et al. 2017; Pereira et al. 2018), we are afraid that we cannot applied the above-mentioned approaches (based on ecological niche modelling and Individual Species Distribution Models -SDM-) because in our study, we are not modelling occurrence but abundance (i.e., count data of Balearic shearwaters). In addition, presence-only SDMs models are based on correlations between species presence and environmental conditions, predicting the environmental suitability for a species, and not their realized distribution, leading to weak relationships between predicted presence and relative abundance (Gomes et al. 2018). By modelling abundance instead of occurrence, we can obtain predictions on real abundance that can provide more relevant information to delineate marine protected areas. In relation to models predicting animal occurrence, few studies have tried to model estimates of relative abundance, even when data are acquired through systematic surveys. In addition, modelling only abundance-given-presence approaches as that in our study have been a rare approach in ecological modelling (Pearce and Boyce 2006). All this further discussion has been included in the Discussion section of the manuscript (lines 343-351). In addition, we have clarified our approach both in the Introduction and Methods section. We have also changed the title of the manuscript, replacing the reference to the “Random Forest” analysis and highlighting the fact that we model abundance data.

**Reviewer #1:** Apart from that, a major weakness of RF, just as in decision trees, is that they cannot extrapolate outside the range of the training data, and therefore great care must be taken when these algorithms are used to predict trends in the long-term, as those expected in the context of climate change. For these reasons, I think authors should attenuate the importance of using this machine learning algorithm in the paper, beginning with removing it from the title.

**Authors:** We agree with the reviewer#1 that Random forest, like other machine learning algorithms, are quite unreliable when extrapolating outside the range of the predictors’ values provided for training. We have removed mentions to future projections (see line 398 and lines 446) and added the specific validity of these models for understanding environmental changes within the range of the training data (see line 476). However, even with this limitation, as it is stated in the text, “trends in abundance related to changes in fisheries and/or in climate affecting the Balearic shearwater distribution can be inspected from a range of latitude and year values over the study period” (see lines 426-429).

**Reviewer#1:** Below I will refer to specific section and lines to provide comment:

As I have already commented, I would recommend changing the title, omitting reference to machine learning on it.

**Authors:** The title has been changed according to the answers given to the reviewer#1 comments above. Following the reviewer suggestion, we have removed “random forest” mention but we have highlighted the use of abundance data.

**Reviewer:** Abstract:

There is a lack of introduction. Please include at least one or two sentences about the general framework before stating what you did address.

**Authors:** Done. Please see lines 29-30.

**Reviewer:** Introduction:

In general, I think the introduction needs a bit of work. Some sentences are not explained well enough. The first paragraph is not very cohesive.

**Authors**: We have now re-written the Introduction section.

**Reviewer**: L49: Should be rephrased. Although I understand what authors mean, these concepts could be explained in a clearer way.

**Authors:** Done.

**Reviewer:** L52-53: I would say seabirds are threatened because they are exposed to multiple threats -most of them related to human activities- as they cross and use multiple habitats year-round, not because they are subjected to many different environmental conditions (to which they are -or should be- adapted). Please rephrase.

**Authors:** Done.

**Reviewer:** L53: I do not understand why you use “however”. I understand that phrase is taking about something completely different from the previous ones.

**Authors:** This sentence has been now totally re-phrased.

**Reviewer:** L54: Reference 3 does not support what is said.

**Authors:** This reference was removed and replaced by Felis et al. 2019.

**Reviewer:** L60: Reference 7 is about passerines. You should provide more references regarding the importance of stop-overs in species of marine or coastal habits.

**Authors:** Done. We have removed this citation and we have replaced it by a new one on seabirds. Please see new citation (Felis et al. 2019). In addition, we have also replaced other citations by specific seabird references (see line 61, Guilford et al. 2009).

**Reviewer:** L62-66. I do not agree with what is stated here. Determining the spatial distribution of seabirds during the migration period is a difficult task because birds they are generally located beyond the reach of the human eye. It is not obvious to me what you mean when saying “surveys of birds are usually limited to specific subsets of the total of the total migratory route”.

**Authors:** We agree with the reviewer and we have re-phrased the sentence accordingly.

**Reviewer:** L64: your statement is false for seabirds. Ring recoveries are not useful for identifying general movement patterns in seabirds, not only stop-over areas.

**Authors:** We disagree with the reviewer. Traditional studies based on ringing recoveries or ocean sightings have proved valuable for identifying very general movement patterns, but fail to discriminate important localities at sea (please see the citation originally provided -Walker et al. 2013- and also Guilford et al. 2009, which is now also cited in the text).

**Reviewer:** L68: accelerometers are not intended to track animal movements but behaviour, so they do not contribute to know at-sea distribution of seabirds.

**Authors:** We agree with the reviewer thus we have removed this mention in the new version of text.

**Reviewer:** L69: I do not agree. Light-level geolocators are the most extensively used devices to track migratory movements of seabirds, and the cost is not a general problem with them. Indeed, geolocators have the advantages of lilted weight, low price and animal-welfare, easy attachment, so high sample sizes are affordable. The real problem is what you explain in the next phrase. Even good sample sizes could be unrepresentative of seabird colony size (Soanes et al. (2013) How many seabirds do we need to track to define home‐range area? Journal of Applied Ecology, 50(3), 671-679; Thaxter, et al. (2017) Sample size required to characterize area use of tracked seabirds. The Journal of Wildlife Management, 81(6), 1098-1109).

**Authors:** We agree with the reviewer thus we have re-phrased the sentence accordingly. In addition, both citations indicated by the reviewer have been included in the new version of the text.

**Reviewer:** L75: references are in wrong format. Moreover, both references do not refer to seabirds, even though you are already focused on seabirds since the first paragraph. Please use more appropriate references (i.e. regarding seabirds).

**Authors:** Done.

**Reviewer:** L76: format of references is wrong.

**Authors:** The format has been now corrected.

**Reviewer:** L77-78: Electronic devices are intended to provide individual tracking and thus get information at individual level. Taking this in mind, they can allow indeed long-term monitoring, and individuals of different species have already been tracked for more than a decade. In the case of seabirds and geolocators, devices can also be replaced annually, so as long as individuals are alive and can be recaptured, long term tracking can be carried out. Last, a major advantage of individual tracking is the fact that you know individual origin (i.e. colony, population). So, the weakness you state for tracking devices is inconsistent. Moreover, the weakness you argue about electronic devices also applied to census surveys, as birds counted at coastal points or vessel surveys are of unknown origin, and even species identification could be difficult for some species (as Balearic and Mediterranean shearwater). Instead of looking for weakness of tracking methods, you should highlight the strength of census methods (e.g. take an overview picture despite unknowing ages or colonies of origin). You should also comment a major weakness of census method for seabirds, which is the impossibility to count birds when they use areas out of the human sight during wintering or migration.

**Authors:** Done, please see lines 77-79.

**Reviewer:** L84-91: I think here you did well in relating the strength of citizen science projects- I would suggest you to combine these lines with the previous paragraph, once removed/resolved the issues I said before. May this reference is also of interest for citation: Coxen, C. L., Frey, J. K., Carleton, S. A., & Collins, D. P. (2017) Species distribution models for a migratory bird based on citizen science and satellite tracking data. Global ecology and conservation, 11, 298-311. Last, you should introduce Trektellen here and comment if some other paper has use this datasource before, as I see this is a really good point of your paper.

**Authors:** We have followed all the suggestions made by the reviewer in this specific comment.

**Reviewer:** L92-96: These lines may be in a different paragraph and be complemented with information about techniques for species distribution modelling. As I already said, many algorithms are available to model and predict species distribution and abundance, and some methods even integrated multiple algorithm. Thus, there is room here to give readers a bit introduction about this issue. I would include information on satellite-derived features as second part in the paragraph, as such features are the predictors used in the models.

**Authors**: We have included additional information on modelling techniques and on the relevance of our modelling approach both in the Introduction and in the Discussion sections. However, for the sake of clarity, we have kept this information after the information on satellite-derived features. See lines 125-131 and also answers to comments above.

**Reviewer:** L100: there is a typo after “shearwaters”.

**Authors:** The comma has now been removed.

**Reviewer:** L108-109: Please provide a reference. Which unused areas?

Done. We referred to unused areas non-detected in the previous studies. See Meier et al. 2015 which is now cited in the text.

**Reviewer:** L114: replace “useful data” by “useful source of data”

Following the other reviewer suggestion, “other useful data” have been replaced by “other records”.

**Reviewer:** L116: replace “these datasets” by “citizen science data”

**Authors:** This sentence has been rephrased following the other reviewer suggestions, thus “these datasets” has now been removed.

**Reviewer:** L118: “most likely pelagic habitats”. This is inconsistent. You said in L112 the species occurs mainly in shallow coastal waters. Also, observation from coast only frequently allow to count birds in coastal waters. So identify pelagic habitats used during migration seems unfeasible.

**Authors:** “pelagic” has been replaced by “marine”.

**Reviewer:** L120: I believe the aim was to evaluate the performance, not to determine it

**Authors:** We agree with the reviewer observation, thus “determine” has been replaced by “evaluate”.

**Reviewer:** L121: replace “seabirds” by “Balearic shearwaters”.

**Authors:** Done.

**Reviewer:** L122: remove “of these and other species”. Also, you could include at the end something like “and discuss the potential of citizen science data for seabird conservation”.

**Authors:** Done.

**Reviewer:** L134-136: I suggest you include this in the next section.

**Authors:** Done.

**Reviewer:** L147: Ref. 32 is related to a different species, remove it.

**Authors:** Done.

**Reviewer:** L148: you should cite this paper, as one of the first describing environmental features to predict the presence of Balearic shearwaters: Louzao, M.et al. (2006) Oceanographic habitat of an endangered Mediterranean procellariiform: implications for marine protected areas. Ecological applications, 16(5), 1683-1695.

**Authors:** Done.

**Reviewer:** L33: this reference is from a symposium in 1993. Please, try to provide more appropriate references. Lot of really good work has been done in the last 20 years with Balearic shearwaters, even addressing the specific question you mention in L10-151. Just two examples:
• Jones, A. R., Wynn, R. B., Yésou, P., Thébault, L., Collins, P., Suberg, L., ... & Brereton, T. M. (2014). Using integrated land-and boat-based surveys to inform conservation of the Critically Endangered Balearic shearwater. Endangered Species Research, 25(1), 1-18.
• Wynn, R. B., Josey, S. A., Martin, A. P., Johns, D. G., & Yésou, P. (2007). Climate-driven range expansion of a critically endangered top predator in northeast Atlantic waters. Biology Letters, 3(5), 529-532.

**Authors:** Done.

**Reviewer:** L151: replace “pelagic and demersal” by “pelagic but also demersal”.

**Authors:** Done.

**Reviewer:** L151: You start the phrase referring to Balearic shearwater’s diet. I guess where you say “but it also feeds” are referring to the species, not the diet. Let’s say: “Balearic shearwater’s diet includes small pelagic but also demersal fish, frequently obtained from trawling discards. The species can eventually feed on plankton and macrozooplankton, specifically krill”.

**Authors:** Done.

Material & Methods.

**Reviewer:**L166: I recommend changing “observatories” by sighting points for clarity.

**Authors:** Done.

**Reviewer:**L171-172: May 1st – August 30th.

**Authors:** Done.

**Reviewer:**L179: Ref. 39 appears two times

**Authors:** We have removed the duplication.

**Reviewer:**L187: I find necessary to provide also a reference on shearwaters, at least a closely related species. Dias, M. P., Granadeiro, J. P., & Catry, P. (2012). Do seabirds differ from other migrants in their travel arrangements? On route strategies of Cory’s shearwater during its trans-equatorial journey. PLoS One, 7(11), e49376.

**Authors:** Done.

**Reviewer:**L187: you say “fluctuations in migration are closely related to changes in food resource”. What are you referring to? Do you mean bird abundance during migration? Do you mean fluctuations in areas used (location) during migration? Do you mean fluctuations in dates? Please clarify.

**Authors:** We referred to bird abundance during migration. This has now been clarified in the new version of the text.

**Reviewer:**L192-193. I find this phrase unnecessary, and the reference provided not appropriate. Papers on seabird research usually indicate. I would suggest rephrasing to something like “We used chlorophyll concentration (Chla, measured in mg.m-3) as a proxy of marine productivity [ref]. We download satellite-based monthly products at 4 km spatial resolution for 2003-2017 period”. Ref. is Wakefield, E. D., Phillips, R. A., & Matthiopoulos, J. (2009). Quantifying habitat use and preferences of pelagic seabirds using individual movement data: a review. Marine Ecology Progress Series, 391, 165-182.

**Authors:** Done.

**Reviewer:**L195-198: This is an important methodological issue. I am going to use this moment to comment my concern about the unbalance in temporal representativity of predictors. You only use/have two years of fishing vessels distribution…I wonder how the unevenly representativity of predictor can affect Random Forest accuracy and predictions….

**Authors:** Discard availability may influence the at-sea distribution of shearwaters. As a proxy of food availability (both discards and fishes) we used information on fisheries. This information was inferred from the distribution of fishing vessels between 2014 and 2015, sourced by the JRC Data Catalogue. This product identifies which are the areas where fishing is more frequent. Vessel tracking data was derived from the Automatic Identification System (AIS), an open source data allowing to analyse the relation between fishing communities and fishing areas at high spatial resolution across Europe. Specifically, data used to build the map consist of 150 million positions from European fishing vessels above 15 m in length. In spite of its limited temporal coverage, the main strength of this dataset is its fine spatial resolution. This proxy on food availability in spatial terms, however, is complemented in our analysis with the high temporal resolution in marine productivity provided by chlorophyll concentration.

**Reviewer:**L202: I find necessary to cite at the end of the phrase “…modulating migratory behaviour” the reference: González-Solís, J., Felicísimo, A., Fox, J. W., Afanasyev, V., Kolbeinsson, Y., & Muñoz, J. (2009). Influence of sea surface winds on shearwater migration detours. Marine Ecology Progress Series, 391, 221-230.

**Authors:** Done.

**Reviewer:**L203: First reference in incorrect format, and also I find this reference no proper here. But again you have many to pick up one:
• Catry, P., Dias, M. P., Phillips, R. A., & Granadeiro, J. P. (2011). Different means to the same end: long-distance migrant seabirds from two colonies differ in behaviour, despite common wintering grounds. PLoS One, 6(10), e26079.
• Dias, M. P., Granadeiro, J. P., & Catry, P. (2013). Individual variability in the migratory path and stopovers of a long-distance pelagic migrant. Animal Behaviour, 86(2), 359-364.
• Dias, M. P., Granadeiro, J. P., & Catry, P. (2012). Do seabirds differ from other migrants in their travel arrangements? On route strategies of Cory’s shearwater during its trans-equatorial journey. PLoS One, 7(11), e49376.
But you can also cite this one that found out quite different results with regard to what you were saying:
• Dell’Ariccia, G., Benhamou, S., Dias, M. P., Granadeiro, J. P., Sudre, J., Catry, P., & Bonadonna, F. (2018). Flexible migratory choices of Cory’s shearwaters are not driven by shifts in prevailing air currents. Scientific reports, 8(1), 3376.

**Authors:** We have added two of the citations suggested by the reviewer supporting our statement as well as the one showing opposite results.

**Reviewer:**L203: Update [29] in Bibliography as this paper is already published.

**Authors:** Done.

**Reviewer:** I find that text in pages 10-11 could be reduced and summarize drastically to ease reading….Yo may included details in Supplementary Material. I think you give too much details about predictors used but could be omitted simply citing references were each predictor has been used before.

**Authors:** Done. See the new Supplementary Material section (specifically, the Supplementary Methods section).

**Reviewer:** Regarding Table 1:
Indicate in the “Total” row that it is the response/target variable.

**Authors:** Following the editor suggestion, response variable has been removed from Table 1 in the present version of the manuscript.

**Reviewer:** Batim: remove year

**Authors:** Done. See also the other non-environmental predictors removed according to suggestions made by the editor.

**Reviewer:** Since the work is based on citizen science, I would expect to find “day of the week” in the table of predictors, as volunteers usually go birding on weekend.

**Authors:** Day of the week should not affect the magnitude of the response variable (abundance of shearwaters) thus we think it should not be included as an additional predictor of shearwater abundance. However, we agree with the reviewer that it could affect the temporal representation of the data. In this sense, we have added a new Figure (see Figure S2 in the Supplementary Material) showing that the datasets considered in our study contain a sufficient sample of observations in different days over the week.

**Reviewer:** Another important detail to me is that first paragraph of Results section should be moved to Material and Methods, as an exploratory analysis is part of methods and drives your next methodological steps. Idem for second paragraph in Results.

**Authors:** We disagree with the reviewer and we think that even if exploratory analysis, these are preliminary results that must be included in the Results section thus we have kept the structure of this section as it was.

**Reviewer:** Another point to highlight in this point is that you do not show any data regarding sampling effort of the citizen science project. The consistency in these projects is key for data quality and even though RF can deal with this issue, it would be informative to see a plot with sampling effort (for example days of observation) at the 123 observatories over time.

**Authors:** As our response variable are daily counts, number of records reported in the Results section is actually the total number of sampling days considered in the analysis. Total number of birds sighted during this sampling days is also reported in the Results section (see also answers to the editor comments above). In addition, regarding the sampling effort over time, we now provide the records conducted in different months over the study period considered in our research, as well as in different days of the week (see Figure S1 and S2 in the Supplementary Material; see also answers to comments above).

Statistical analysis

**Reviewer:** I recommend starting this section clearly indicating the structure of your data matrix, i.e. clearly defining whether each row is an observatory point, a session (i.e. specific day) from an observatory point, or whatever.

**Authors:** Done.

**Reviewer:** L60: I would recommend citing references already published. If it is not possible, at least provide some extended explanation to understand your choice without the need to find a not yet published paper.

**Authors:** Unfortunately, the manuscript cited is still under review thus we cannot provide the reference to a published paper. However, following the suggestion of the reviewer, we have provided an extended explanation of the results obtain in the mentioned research. Please, see lines 216-233.

**Reviewer:** L243: It is not clear to me why you log-transformed the abundance data. Please clarify it.

**Authors:** As it is stated in the text, abundance of shearwaters are count data that follow a Poisson distribution. Prior to build our models we log-transformed the abundance data in order to improve the accuracy of the estimated effects reducing the potential bias and increasing the predictive power of the model. We have included this clarification in the new version of the manuscript.

**Reviewer:** L246-256: You should provide more details about tuning parameters of RF. Please clearly state the total sample size in the data matrix used as input in the models (number of rows, number of columns), the number of trees you grew up, the number of predictors you used for each tree, and the number of samples you select in each tree. It is not clear if you are using cross-validation, please try to clarify your explanations in L253.

**Authors:** Sample size of the shearwater observations (i.e., rows in the data matrix) is provided in the Results section (see lines 278-282). As in any usual regression analysis, the number of columns in the data matrix matched the number of predictors (see Table 1). All the trees were built using the total set of predictors described in Table 1. We assessed the performance of the models based on the Out- of-bag (OOB) error. The random forest model was trained using bootstrap aggregation, where each new tree was fit from a bootstrap sample of the training observations *z_i_=(x_i_,y_i_)*. The OOB error is the average error for each *z_i_* calculated using predictions from the trees that do not contain *z_i_* in their respective bootstrap sample (Hastie et al. 2009). This allows the model to be fit and validated whilst being trained, thus no additional cross-validation was required. Number of trees assessed ranged between 100 and 500. For tuning the parameters of the model, we applied a grid search method, thus we evaluated the model over different combinations of parameters included in the grid (values ranging between 1-15 at one unit intervals for mtry parameter).

For the sake of clarity, we have re-written this section and all this explanations and the above-mentioned citation have been now included in the Methods section.

T. Hastie, R. Tibshirani and J. Friedman, “The Elements of Statistical Learning 2^nd^: Data Mining, Inference, and Prediction”, p592-593, Springer, 2009.

**Reviewer:** L249: You refer RMSE without defining its meaning before. Please do explain briefly to the readers why you used Root Men Square Error for parameter tuning.
I would expected to see the confusion matrix (predicted vs observed) to generally evaluate accuracy of RF models. Could you include it?

**Authors**: RMSE (Root Mean Square Error) is the average difference between the observed known values of the outcome and the predicted value by the model. It is not solely a measure for parameter tuning but, on the whole, a usual measure used for assessing the accuracy of random forest regression models. The lower the RMSE value, the better the model. Further clarifications regarding this issue have been now included in the Methods section.

Regarding confussion matrix, we cannot provide it because we are modelling abundance (i.e., count data; see new lines added: 192-193), thus we are not calibrating random forest classification trees (models where the response variable is binary) but random forest regression trees (models where the response variable is continuous). Therefore, as it is stated in the text, we inferred the correspondence between observed and predicted values from RMSE.

**Reviewer:** L257: A bit confusing...May be rephrase: “Relative importance of the variables used for predicting shearwater abundance…”

**Authors**: Done.

**Reviewer:** Regarding Variable Importance, considering most readers of this paper will be ecologists from outside computing science, I would recommend to use a metric (and plots) easier to follow and understand. Ranking variables with the Gini Index or Mean Decrease Accuracy and showing a barplot illustrating the ranking would be much more understandable than plots provided by randomForestExplainer. In my opinion, when predictors overlap in the ranking the visualization provided by this R package is not so effective, as shown in figures 3a and 3b.

**Authors**: As we explained above, we are modelling random forest regression trees, thus we cannot apply the measures suggested by the reviewer, since Gini Inces and Mean Decrease Accuracy are measures of node impurity in classification Random Forest. In addition, these ranks rely on the choice of a performance measure, although measures of prediction performance are not unique. To solve this, Ishwaran et al. (2010) developed a method called minimal depth (MD) that simply determines variable importance by the position of the variables in the decision trees and thus, is only based on the decision tree structures. The idea is that, variables that tend to split close to the root node should have more importance in prediction. We think that our approach, even more complex, provides a better understanding of the relative variable importance and, therefore, it should be kept, since it is not only a matter of plotting but a matter of the way we quantified the variable importance.

Results

**Reviewer:** As I said before, I think the first and the second paragraph of Results section should be moved to Material and Methods, as an exploratory analysis is part of methods and drives your next methodological steps.

**Authors**: See comment above. In our experience (as researchers writing papers and as reviewers of scientific publications) even if they are exploratory, all analysis conducted are expected in the Results section, since the Methods section is only a description of the procedures that were applied to obtained the results. Therefore, we decided to keep the structure of this section as it was.

**Reviewer:** L276-277: The way this sentence is written is somehow confusing, as you only run two models (pre & post-breeding migration), but from the text it seems as you run a lot. As building a RF model implies using different training data subsample, I recommend removing this from the sentences “among the models built with different training data subsamples”.

**Authors**: In fact, as it was stated in the text, any random forest model is trained using bootstrap aggregation, where each new tree is fit from a bootstrap sample of the training observations (i.e., bagging procedure). Therefore, when calibrating a random forest model, we are actually running multiple iterative trees. Therefore, we can derive errors (and confidence intervals) from the different trees calibrated (see also answer to the comments above).

**Reviewer:** L308-310. This result could be due to a temporal bias in sampling effort from observatories. You should show, at least, the number of days of observation per Latitude and Year to support this result.
Fig. 5, figure caption: I think it is more properly as: “Predictions of Balearic shearwater spatial distribution and abundance during migration”

**Authors**: Since the study period is 13 years (2005-2017) and the study area contains 40,337 spatial cell units at the modelled resolution, it is not feasible to easily show together annual sightings per location and year, neither in a map nor in a table. However, the proper spatial representativeness of the data used in this study is now reported in Figure 1 as graduated symbols representing the number of sightings (see also comments of the editor) per location and migration period (pre-breeding and post-breeding). In addition, sightings per year and month are reported in Figure S1 in the Supplementary Material.

Regarding the Fig. 5 caption, we have mostly followed the suggestion made by the reviewer#1 and it is now as following: “Predictions of Balearic shearwater spatial distribution of abundance during migration”.

Discussion

**Reviewer:** L349-352: I understand Big Data is trending topic…But this paper is really far from using Big Data, so I recommend to avoid the use of this term.

**Authors**: In spite of the relatively reduce data set finally analysed (about thousands of shearwater records), open data sources managed during the analysis and later predictions conducted in our research (mostly NCEP reanalysis -weather predictors) contain more than 1,250,447 rows, thus we think it is worthy of being mentioned as “Big Data”.

**Reviewer:** L358: My suggestion: “allow to identify general spatio-temporal patterns as data come from different individuals belonging to several populations”.
You should discuss in this part the weakness of data coming from volunteering programmes, such as temporal gaps, uneven sampling effort in space and time, etc. particularly in L353 where you indicate “its characteristic noise” but do not explain anything more.

**Authors**: We have followed the suggestion made by the reviewer and the text has been changed accordingly. In addition, we have also discussed about the weakness of citizen science data and we have added a few citations to support our discussion.

**Reviewer:** L361-365: This phrase is too long, 5 lines…. Please rephrase or split it.

**Authors**: Done.

**Reviewer:** L368: RF are not good to forecast out of the range of input data, so future projections based on this could be unreliable… depending on input data. Moreover, I think this issue (I mean future projections) is out of the scope of this paper.

**Authors**: We agree with the reviewer and we have removed any mention to future projections derived from the models.

**Reviewer:** L371: ref [74] is about ducks. I would say there are more appropriates references to cite here related with seabirds.

**Authors**: We have followed the suggestion made by the reviewer and the original citation has been replaced by a more suitable one (Tsikliras et al. 2019).

**Reviewer:** L379: format of references incorrect.

**Authors**: The citation was misplaced thus we have removed it in the new version of the manuscript.

**Reviewer:** L382: Be consistent with therm used. Replace volunteer data by scitizen science data.

**Authors**: Done.

**Reviewer:** L393: what do you mean to “larger variability observed”? which result support this?

**Authors**: We referred to the wider confidence intervals in the model accuracy measures. This has now been clarified in the text.

**Reviewer:** L395-397: This phrase is not clear. Please rephrase it.

**Authors**: Done.

**Reviewer:** L422: this is related to some clarifications needed that I mentioned before.

**Authors**: We meant “when variability in accuracy of the predicticted abundance is larger”. This has now been clarified in the text. See also comment above.

**Reviewer:** L433-448. I found Conclusions section clear and nice

**Authors**: Thank you!

Bibliography:

**Reviewer:** There are typos spread over the section.

**Authors**: We have corrected the typos throughout this section.

6. PLOS authors have the option to publish the peer review history of their article ([what does this mean?](https://journals.plos.org/plosone/s/editorial-and-peer-review-process#loc-peer-review-history)). If published, this will include your full peer review and any attached files.


If you choose “no”, your identity will remain anonymous but your review may still be made public.


**Do you want your identity to be public for this peer review?** For information about this choice, including consent withdrawal, please see our [Privacy Policy](https://www.plos.org/privacy-policy).

Reviewer #1: No


[NOTE: If reviewer comments were submitted as an attachment file, they will be attached to this email and accessible via the submission site. Please log into your account, locate the manuscript record, and check for the action link "View Attachments". If this link does not appear, there are no attachment files to be viewed.]


While revising your submission, please upload your figure files to the Preflight Analysis and Conversion Engine (PACE) digital diagnostic tool, <https://pacev2.apexcovantage.com/>. PACE helps ensure that figures meet PLOS requirements. To use PACE, you must first register as a user. Registration is free. Then, login and navigate to the UPLOAD tab, where you will find detailed instructions on how to use the tool. If you encounter any issues or have any questions when using PACE, please email us at [figures@plos.org](mailto:figures@plos.org). Please note that Supporting Information files do not need this step.

*In compliance with data protection regulations, you may request that we remove your personal registration details at any time.* [*(Remove my information/details)*](https://www.editorialmanager.com/pone/login.asp?a=r)*. Please contact the publication office if you have any questions.*
