# Supplementary material for: Citizen science for predicting spatio-temporal patterns in seabird abundance during migration
Source: PLoS One. 2020 Aug 14;15(8):e0236631. doi: 10.1371/journal.pone.0236631 (PMC7428152; doi:10.1371/journal.pone.0236631)
Supplement: Attachment — Submitted filename: answers_to_reviewers_may2020.docx [file pone.0236631.s004.docx]

1^st^ June 2020

PONE-D-19-29580R1

Citizen science for predicting spatio-temporal patterns in seabird abundance during migration

Vitor Hugo Rodrigues Paiva
Academic Editor
PLOS ONE

Dear Mr. Rodrigues,

We sincerely thank you for allowing us to resubmit our paper. Following your instructions, we resubmit the manuscript titled “Citizen science for predicting spatio-temporal patterns in seabird abundance during migration” (PONE-D-19-29580R1), after fully addressing all the concerns raised by the reviewers.

Changes done throughout the text have been recorded, via track-changes, in a document enclosed with this re-submission. Please find below the detailed explanation of the modifications we have made in response to the concerns raised. A copy of the document with the *track changes* feature turned off is also attached. Lines indicated below referred to the document with the *track changes* feature turned on.

We hope that you will find this revised version suitable for publication in *PLOS ONE*.

Yours faithfully,

Beatriz Martín on behalf of all the authors

PONE-D-19-29580R1
Citizen science for predicting spatio-temporal patterns in seabird abundance during migration
PLOS ONE

Dear Dr Martin,

Thank you for submitting your manuscript to PLOS ONE. After careful consideration, we feel that it has merit but does not fully meet PLOS ONE’s publication criteria as it currently stands. Therefore, we invite you to submit a revised version of the manuscript that addresses the points raised during the review process.

We would appreciate receiving your revised manuscript by Jun 07 2020 11:59PM. When you are ready to submit your revision, log on to https://www.editorialmanager.com/pone/ and select the 'Submissions Needing Revision' folder to locate your manuscript file.

If you would like to make changes to your financial disclosure, please include your updated statement in your cover letter.

To enhance the reproducibility of your results, we recommend that if applicable you deposit your laboratory protocols in protocols.io, where a protocol can be assigned its own identifier (DOI) such that it can be cited independently in the future. For instructions see: <http://journals.plos.org/plosone/s/submission-guidelines#loc-laboratory-protocols>

Please include the following items when submitting your revised manuscript:

- A rebuttal letter that responds to each point raised by the academic editor and reviewer(s). This letter should be uploaded as separate file and labeled 'Response to Reviewers'.
- A marked-up copy of your manuscript that highlights changes made to the original version. This file should be uploaded as separate file and labeled 'Revised Manuscript with Track Changes'.
- An unmarked version of your revised paper without tracked changes. This file should be uploaded as separate file and labeled 'Manuscript'.

Please note while forming your response, if your article is accepted, you may have the opportunity to make the peer review history publicly available. The record will include editor decision letters (with reviews) and your responses to reviewer comments. If eligible, we will contact you to opt in or out.

We look forward to receiving your revised manuscript.

Kind regards,

Vitor Hugo Rodrigues Paiva
Academic Editor
PLOS ONE


Reviewers' comments:

Reviewer's Responses to Questions

**Comments to the Author**

1. If the authors have adequately addressed your comments raised in a previous round of review and you feel that this manuscript is now acceptable for publication, you may indicate that here to bypass the “Comments to the Author” section, enter your conflict of interest statement in the “Confidential to Editor” section, and submit your "Accept" recommendation.

Reviewer #1: All comments have been addressed

Reviewer #2: (No Response)

2. Is the manuscript technically sound, and do the data support the conclusions?

The manuscript must describe a technically sound piece of scientific research with data that supports the conclusions. Experiments must have been conducted rigorously, with appropriate controls, replication, and sample sizes. The conclusions must be drawn appropriately based on the data presented.

Reviewer #1: Yes

Reviewer #2: Partly

3. Has the statistical analysis been performed appropriately and rigorously?

Reviewer #1: Yes

Reviewer #2: No

4. Have the authors made all data underlying the findings in their manuscript fully available?

The [PLOS Data policy](http://www.plosone.org/static/policies.action#sharing) requires authors to make all data underlying the findings described in their manuscript fully available without restriction, with rare exception (please refer to the Data Availability Statement in the manuscript PDF file). The data should be provided as part of the manuscript or its supporting information, or deposited to a public repository. For example, in addition to summary statistics, the data points behind means, medians and variance measures should be available. If there are restrictions on publicly sharing data—e.g. participant privacy or use of data from a third party—those must be specified.

Reviewer #1: Yes

Reviewer #2: No

**Authors:** All the environmental predictors used in the present study are freely available from the websites of the different data sources indicated in Table 1. Moreover, the abundance of shearwater analysed in this study can be obtained after request from eBird (<https://ebird.org/>). This information is now explicitly indicated in the Acknowledgements section.

5. Is the manuscript presented in an intelligible fashion and written in standard English?

PLOS ONE does not copyedit accepted manuscripts, so the language in submitted articles must be clear, correct, and unambiguous. Any typographical or grammatical errors should be corrected at revision, so please note any specific errors here.

Reviewer #1: Yes

Reviewer #2: Yes

6. Review Comments to the Author

Please use the space provided to explain your answers to the questions above. You may also include additional comments for the author, including concerns about dual publication, research ethics, or publication ethics. (Please upload your review as an attachment if it exceeds 20,000 characters)

Reviewer #1: I congratulate the authors for changes accounted over the manuscript, which is now much easier to follow, readable and understandable than the previous version. In my opinion they resolved successfully most of the comments done in the first submission. Nevertheless I have some comments on this new version. Despite most of them are minor comments, I really do think authors should amend them to improve the manuscript for publication.

My comments are detailed below. Line numbers correspond to those of the version with “Tracking changes” activated.

L36: I suggest "Random Forest regression models"

**Authors:** Done.

L54: ecosystemS, “s” in plural.

**Authors:** Done.

L65: have been received (remove “been”)

**Authors:** Done.

L74: I suggest replace “limit” by other word such as “undermine”, “affect”.

**Authors:** Done.

L90: I suggest: species migratory range due to the variety of migratory strategies at colony- and individual levels.

**Authors:** Done.

L98: replace “Therefore” by other term to avoid starting in the same way than previous sentence, it would make reading more fluid.

**Authors:** Done.

L98-99: I suggest: even providing very detailed information on INDIVIDUAL SEABIRDS’ movements, have a limited ability…

**Authors:** Done.

L101: My suggestion: Census methods, in contrast, can provide a VALUABLE overall picture, even though they do not allow to detect birds when they use areas out of human sight.

**Authors:** This was a correction made by other reviewer in the previous reviewing round. What we mean here is that census data do not allow to identify the colony or the age of the individuals. We have slightly changed the sentence in order to clarify it for the reader. Please see line 82.

L106-131. Just another suggestion to ease reading. You used “however” and “in this sense” repeatedly to start sentences. May be you can replace some of them for readability.

**Authors:** Done.

L130: as you say “almost unexploited” I would expect to see at least one reference using Trektellen. If there are no publications, it may be better to say “have remained unexploited by the scientific community to the best of our knowledge” or something similar.

**Authors:** Done.

L143: Replace “geolocator archival tags” by “geolocation archival tags”, which is the correct form.

**Authors:** Done.

L167: may provide a useful records (delete “a”)

**Authors:** Done.

L178-179: the correct name does not include “Individual”, just “Species Distribution models”.

**Authors:** Done.

L263: How many sighting points from eBird?

**Authors:** Sighting points are not fixed in eBird observations so the number of observations is the number of sighting points. We have now clarified this point (see line 183).

L275: Why “Environmental” with capital letter?

**Authors:** Done.

L277: I suggest to put a comma before “thus” to ease reading. This advice also applies to many other "thus" along the manuscript.

**Authors:** Done. We have changed this throughout the manuscript.

L288: Caption of Table 1. For consistency you should say “Description of environmental predictors.”.

**Authors:** Done.

L393: Classification and Regression TreeS, add “s”.

**Authors:** Done.

L414-417: Honestly, to the best of my knowledge there is no need to apply any kind of transformation on the response variable to run Random Forest models. Conversely to GLMM and other parametric methods, random forest do not have any kind of assumption about distribution of data or residuals, so that count data adjust to a Poisson distribution is irrelevant here. The same about the predictive power of the model, it will not change because of transforming the response variable. A different thing is to transform predictors applying standardization, but that is different and is not what was done here. To make sure about my previous knowledge, I did a little research trying to find support to your statement, with no luck. May be I am wrong, so I sincerely encourage the authors to provide a reference supporting that transforming the response variable improves accuracy and increases predictive power of Random Forest models. If you do not find such support, I think the log transformation could be simply justified to improve interpretability of data -which (to me) should be enough-, but not to improve accuracy and predictive power of Random Forest.

**Authors:** The reviewer is right in relation to the lack of assumptions in the distribution of the data in Random Forest models. However, when we use an approach based on decision trees such as Random forest where data partitioning is applied, we can obtain better results if we work with a dependent variable homogenously distributed, because the dispersion increases as long as the variable increases. We cannot provide a reference but we have provided our own results (see lines 255-263).

L515: Regarding Fig. 4. Please (1) indicate in legend that values are in ln, or back transform them to the actual scale, (2) change year to factor so in the X axis years do not appear with decimal digits.

**Authors:** Done. We have also clarified the scale of the reported predictions in the legend of Figure 5 and Figures S4-S6 in the Supplementary material.

L532: Caption of Figure 5. I think “spatial distribution of abundance” can be confusing for readers. My suggestion to the entire caption to ease readability: “Predicted abundance of Balearic shearwater across its distribution range during migration. Colour gradient indicates the percentage of the maximum predicted abundance across the study area on the fifteenth of the month, from May to December, from environmental conditions (see Table 1) occurred in year 2017."

**Authors:** We have accepted the suggestion made by the reviewer. Please see the new Figure legend.

L553: “Our results showed (…) successfully applied to describe the migratory distribution (AND ABUNDANCE?) of seabird species accurately.”

**Authors:** Done.

L579: increaseD, add "d"

**Authors:** Done.

L587-596: “In contrast…(…) in the long-term.” Phrase too long, more than five lines…hard to read. Please split it.

**Authors:** Done.

L636-637: “For instance, locations with tailwinds, high abundance level and low chlorophyll concentration”, reorder, as in the next sentence, to: For instance, locations with high abundance level, tailwinds, and low chlorophyll concentration”.

**Authors:** Done.

L650: “But the major achievement of our modelling attempt is the high temporal resolution that we achieved with our models”. May be better to rephrase this phrase to avoid tongue twisters….for instance substituting “achievement” by accomplishment, and “modelling attempt” by analysis (in fact, as you already performed the modelling, it is no longer an attempt, isn’t it?).

**Authors:** Done.

L676: “the two migration movements”. I suggest to change this by “the two migratory periods” for clarity, and for consistency (see your lines 266-267).

**Authors:** Done.

L711: In relation to successful approaches developed to identify marine Important Bird Areas, instead of reference [69] I find much more appropriate and relevant to cite the work developed by SEO/BirdLife, a fortiori, in a work regarding Balearic Shearwater. Either the working-example paper https://www.sciencedirect.com/science/article/abs/pii/S0006320711004745 or the book Arcos, J. M. (2009). Áreas importantes para la conservación de las aves marinas en España: Important areas for the conservation of seabirds in Spain. Sociedad Española de Ornitologia (SEO/BirdLife).

**Authors:** Done.

Reviewer #2: First of all, I’d like to congratulate the authors for the study. The study, definitely, is interesting and relevant as it is aiming at (1) a better understanding of the migration pathways of a highly threatened seabird species whose general knowledge present several gaps, and (2) deal with citizen-science large data banks. Such data banks are very useful and studies that aids in a better understanding of how to analytically deal with citizen science in order to provide biologically and ecologically sound information are always welcome. I have no doubt that this study deserves attention and have potential to make an important contribution on those two topics I mentioned above. Therefore, I did a careful reading of the manuscript and checked with attention the critics of the previous reviewers and the authors’ response. I know the reviewing process can be daunting sometimes, particularly when a first round of reviews have already been done, and then a new reviewer comes in and makes further critics that haven’t been there before. Unfortunately, I think that is going to be my role here. In my view, the main fragilities of this study are: the details of the analytical approach need to be better presented; presentation of results needs to be reviewed, several results would be better presented if figures were made differently and/or a different set of statistical outputs of the RF models were used; and, finally the most critical issue, I ask authors to reconsider using Lat and Long as factors in the modelling, based on figure 3, both Lat and Long are the variables that explained most of models’ power of prediction – what is the consequence of that to the findings? If authors think the use of LatLong is justified, they should explain it carefully in methods and discuss it more deeply. But for me, it is clear on Fig3 that LatLong reduced considerably the importance of the environmental variables and the model is mostly predicting spatial occurrences than habitat use. Below a list of the detailed comments that authors should address.
I ask patience for the authors and hope they understand the comments and critics aims for improving the final manuscript. Have a good review, looking forward to read the reviewed version!

**Authors:** We have followed the suggestions and the recommendations made by the reviewer in relation to the Methods and Results presented in the study, please see comments below. Regarding the longitude and latitude as predictors because, we must have in mind that we are not predicting the static distribution of the shearwaters as a particular time snapshot, but their occurrence along the migration period. Apart from environmental cues such as food availability, migratory birds can more or less rely on photoperiodic cues and/or endogenous rhythms to initiate their migration. Longitude, latitude and date variables allow us to include in the models the endogenous rhythm of the bird. As we can see from the variable importance in the models, whereas food availability seems to be a more important triger of post-breeding migration, the internal rhythm of the bird appears to be more important during the pre-breeding period. For further details, please see comment below and new lines 210-228.

L52 – Seabirds instead of “marine birds”

**Authors:** Done.

L54 – I suggest deleting “migrate” from this sentence. Start next sentence with something like “migratory seabirds are particularly susceptible to a large number of stressors given the variety of habitats they use throughout their year-cycle…”

**Authors:** Done.

L60- Suggest rephrasing to “Stopovers are key sites; conditions experienced by seabirds in stopover can affect individual survival throughout migration and drive population dynamics” or some similar idea.

**Authors:** We have mostly followed the reviewer suggestion. However, since conditions in stopovers may affect survival beyond the migratory journey due to carry-over effects, we have slightly changed the re-phrasing (see lines 61-64). See also the correction made by the reviewer #1 above.

L76 – “and in relation to specific individual traits” this is a bit “loose” within the sentence. I suggest you describe which are those individual traits, or delete this part of the sentence. For instance, in the start of the sentence “This is particularly true for long-lived seabirds with a defined set of traits such as….. which have a great capacity… “

**Authors:** By “traits” we meant sex, age, breeding colony. This has now explicitly written in the text.

L96 – Rephrase “Prominent electronic citizen science data banks includes eBird and trekellen xxxx, two biodiversity…”. Additionaly, why they are prominent? Cited studies compared them with other databanks? Maybe more popular, commonly used, or else…?

**Authors:** They are prominent in number of users. We have clarified this point in the text.

L104 - Birdlife factsheet indicates several parameters explaining why this shearwater is critically endangered and why actions to improve knowledge on population trends are urgent, but I did not find any reference to the species being one of the most threatened species of seabirds. Maybe rephrase emphasizing that information from [26] allows placing this species as one of the most threatened seabirds in the world, as possibly it is.

**Authors:** Done.

L151. Is it possible to plot the position of known breeding colonies of the species?

**Authors:** We think that it is not a good idea to show the breeding colonies in the map because only a few main colonies are known for these species and the information would not be an overall picture of the breeding range. However, following the reviewer suggestion and according to the publication below, we now show both the breeding and the non-breeding range for the species in Figure 1.

José Manuel Arcos: International species action plan for the Balearic shearwater, *Puffinus mauretanicus*, SEO/BirdLife & BirdLife International, 2011,

Line 170. This is key: correctly identified when sighted. Some seabirds at sea are very difficult to distinguish; therefore the use of data from citizen science for study difficult-to-identify species should be careful. I hope that it is acknowledged in the discussion.

**Authors:** Actually, it was mentioned in the Discussion section (see lines 426-431; and line 516).

L183. Data from 2005 to 2017 was used because it is the most representative period in terms of sampling. 2018 was excluded because it does not have a homogeneous sampling throughout the year. Mention it here.

**Authors:** In fact this point had been indicated just in a couple lines below in the previous version of the manuscript: “However, to ensure the maximum seasonal representation of the dataset to be analysed (see Figure S1 in the Supplementary material), from this total sample of birds, we only modelled data collected during the migration period from 2005 to 2017 (see Results)”. Please see lines 188-191.

L191. It seems you used year and geographical position as factors in your model (by seeing figure 3). It is not described in methods how those variables entered the model. I would not recommend using Lat and Long as factors in this case, and I really think it doesn’t help your study at all (if it does, please provide an explanation). Lat and Long are not environmental variables used by the birds, and given that both were the most influencing variables in the model, it is likely the models outputs are predicting the occurrences rather than the relation between abundance and environment. Don’t you agree? If this is true, the model should be run again without lat and long as factors. You could, a posteriori, use a probability of occurrence based on latlong to filter the predictions like Hindell et al. (2020; DOI 10.1038/s41586-020-2126-y) did.

**Authors**: Latitude and longitude were included as discrete variables in the models (this is now explicitly indicated in line 212). We have included latitude and longitude as predictors because we are not predicting the static distribution of the shearwaters as a particular time snapshot, but their occurrence along the migration period. Below we provide a detailed explanation. Depending on the species, birds can more or less rely on photoperiodic cues and/or endogenous rhythms to initiate their migration. Therefore, although migration decisions in shearwaters are partially dictated by food availability, the endogenous programme of the bird should make it to instinctively move into the north as long as the post-breeding season progresses. Date, longitude and latitude variables allow us to include in the models the endogenous rhythm of the bird. In addition, as we mentioned in the Introduction section, breeding and wintering grounds of different shearwater populations differ. Therefore, longitude and also latitude can be indirect proxies of the effects that different wintering sites, different length of the route and different en-route environmental conditions may pose to different migrant birds. These effects are not possible to be quantified in detail from citizen datasets since, in contrast to ringing or electronic tracking devices records, because they do not provide information on the particular origin and destination of the observed bird. On the other hand, and most important, we cannot ignore that we are not predicting merely habitat use but modelling birds which are in movement. In this sense, the use of these additional predictors also allows to incorporate the effects of the complex interactions emerging between predictors, such as latitude, date and temperature. For instance, for a migrating shearwater it has not the same meaning, in terms of migration decisions, to experience 25°C in the Mediterranean Sea in mid-February than 25°C in the UK coasts in mid-August. Similarly, due to different rates of change across space among different bird populations, interactions between predictors and “latitude”, and between “year” and “latitude” may allow to quantify both the spatial and temporal heterogeneity in the migratory responses. We have now briefly clarified all these facts in the new version of the manuscript (please see lines 212-226).

L193. In practical terms, zero was suppressed, right?

**Authors:** The reviewer is right. We have now clarified this point in the text (please see line 199).

L197 – “For a detailed description of the set of predictors included in the models, please see the Supplementary Material.” Instead of this, you could add in line 195 “…predictors (Table 1, Supp. Material).” The references in the supplemental material are the same from the main text? Shouldn’t sup. material have its own reference list?

**Authors:** Done. The sentence has been removed and we have created a new specific section of references in the Supplementary material.

L211. Where exactly in results? Reference to supp. material.

**Authors:** Done.

L212-221. I didn’t understand this section. You applied a single machine-learning based technique (random forest), right? You start the sentence saying it in plural. Then you presented a series of models and an equation, apparently presented in three studies [43,44,45]. If this is true, this paragraph could be simplified to saying that among a handful of techniques, RF was identified as one of the most accurate in at least three studies based on comparison of RMSE. The reader can check then the studies that you cited.

**Authors:** Actually, as the reviewer suggests, this assessment was only cited in the first version of the manuscript. This paragraph was added in the previous reviewing round after request of one of the reviewers, since the cited work remains yet unpublished (it is under review). For this reason, we have decided to keep the further explanation regarding the selection of the modelling approach.

L242 – 248.
This is not clear and needs further explaining. You used bootstrap to calibrate the model, the algorithm stopped when the best “solution” was reached? That’s why you have a variable number of combinations (1-15)? It is also usual to run a fixed number of trees and check the increase in accuracy, and a posteriori, select the trees that produced increasing accuracy without substantial overfit. That’s why you had a variable number of trees between 100 to 500? I imagine you used 500 threes for all the possible variable combinations and used the approach I described to select the best solutions, the minimum number of trees required to achieve that was 100. Is it right? Or I completely misunderstood?
The RMSE is calculated over the OOB-error? It did not seem that you did it. In my opinion, a plot showing OOB vs RMSE (or AUC, or other…) over all iteration trees could provide such information and justify why there is a variable number of trees used in the final and averaged abundance output (I briefly checked the packages you used, there are some ways of doing that: http://topepo.github.io/caret/model-training-and-tuning.html). If this is not what you did and I misunderstood, please provide alternative explanations.

**Authors:** We think the reviewer confuse the machine learning technique applied. Bagging is the application of the Bootstrap procedure to a high-variance machine learning algorithm, typically decision trees. And Random Forest is an improvement over bagged decision trees, consisting of a large number of individual decision trees that operate as an ensemble. As it is stated in the text (see lines 267-269), any random forest model applies bagging (i.e., bootstrap aggregating) to sub-sample the data that are used for training. Please, see also comment below about the difference between boosting and bagging techniques.

As it is described in the Methods, we are following a usual Random Forest regression analysis so the RMSE is calculated over all the trees in a particular model over the bagging procedure. In Random forest each model is calculated through a bagging procedure (i.e., by building multiple different decision tree models from a single training data set by repeatedly using multiple bootstrapped subsets of the data and averaging the models, see lines 267-269). OOB error is derived from this bootstrap process. When bagging decision trees the number of samples and hence the number of trees is a parameter to be selected (see lines 278-279). Therefore, the algorithm increases the number of trees on run after run until the accuracy begins to stop showing improvement. We have now clarified this point in the text (please see lines 278-282). On the other hand, random forest model has hyperparameters that should be tuned to avoid overfitting. The previous is now explicitly clarified in the text (lines 273-274). Therefore, as it was stated in the text, to determine the parameter values offering the best fit, we specified a set of tuning values to be tested during the calibration of the models. That is why we have a number of combinations (1-15). Specifically, we applied a grid search method, thus we evaluated the model over different combinations of parameters included in the grid (values ranging between 1-15 at one-unit intervals). We must have in mind that RMSE is the average difference between the observed known values of the outcome and the predicted value by the model (see lines 243-244). Therefore, the best fit parameter combination is then asseses by means of the RMSE of the models (see new lines 282-284 explicitly explaining the later point).

We could not apply AUC (Area Under the Receiver Operating Characteristics -ROC- Curve) because this measure is only applicable to classification trees but not to regression trees, as it is our approach. That is the reason why we chose RMSE metric.

L272. Order of the figures was quite confusing… figure 1 was the last in the PDF… putting the legends with the figures instead of merging it in the text would facilitate reviewing. I hope authors consider it in further revisions or submissions.

**Authors:** we will take care of the figure ordering when re-submitting the manuscript. However, the inclusion of the legends throughout of the text is a requirement of PLOSONE to indicate the suggested position of the Figure within the text.

L273. You said in methods that you removed correlated variables, and in the end, you used all variables because no variable was correlated. Change it in methods.

**Authors:** Done, see lines 232-235.

L277. Figure 2 points out a small variability on power of prediction based on R2 values, more or less between 0.49 and 0.53, and slightly more accurate predictions during RFPost, as mean error was lower. It is not clear to me how it indicated substantial variation among models. Please explain. Further information is also required: are those results from all the iterated trees?

**Authors:** The differences between pre-breeding and post-breeding models were measured as the lagged and iterated differences over the model resamples. This sentence was initially included in the Result section, but we have now moved it into the Methods. On the other hand, as it is stated in the text, the variation among models built with different training data subsamples is measured as the 95% confidence interval in the used metrics (RMSE and R2). We have now qualified this point in the text. Even if the variability seems small, according to the Results (see lines 328-338), differences in RMSE between pre- and post-breeding models are statistically significant.

L279-281. Please explain what lagged-iterated differences mean and how they were calculated. Is this a lag between iterations or annual variability? Check next comment.

**Authors:** As it is stated in the text, “the differences between pre-breeding and post-breeding models were measured as the lagged and iterated differences over the model resamples”, so the differences are measured over the model resamples, as the difference between a resample and the previous one in an iterative way. We have now included this clarification in the text (lines 328-329).

L299. Year entered as a factor? Or models were run separately for each year? Methods were not clear on how annual variability was used in the models. If year entered as factor in the analysis, how did you deal with fisheries time-coverage being different then the species data? You probably used fishing as a fixed non-dynamic variable. How you justify that?

**Authors:** Year, as well as date, longitude and latitude, entered in the models as discrete variables predicting shearwater abundance. We have now clarified this point (see comment above and lines 210-2132).

L306. What is minimum depth? It is crucial to understand figure 3. An alternative way (more straightforward, in my opinion) of analyzing variable contribution is to plot the change in accuracy when the variable is absent from the model. Seems that Caret package has a standard function to do that: ‘varImp’.

**Authors:** Minimal depth is a method developed by Ishwaran et al. (2010) that determines variable importance by the position of the variables in the decision trees so the importance is based on the decision tree structure. The idea is that variables that tend to split close to the root node should have more importance in prediction. We really think that our approach, even more complex than other simple measures of prediction performance, provides a better understanding of the relative variable importance and, therefore, it should be kept. To help the reader understanding the approach, we have included additional information on the way that minimal depth is measured (see lines 298-302).

L312. Not sure whether figure 4 contributes to the overall results. It could be placed on the suppl. Material. How annual variability was used in the model is not clear either.

**Authors:** We disagree with the reviewer regarding the importance of Figure 4. The results on the interaction between latitude and year variables (which is shown in Figure 4) allow to determine whether there is a spatial trend in abundance over the period. This is explained in lines 340-342. This fact has been previously discussed for this and other seabird species in other research studies, see the Discussion section, and it is relevant both for understanding the effects of climate change and to delimit marine areas (see the Discussion section). For these reasons, we have kept Figure 4 within the main text of the article. Regarding annual variability, as it was stated in the text (see line 213), interannual variability is measured through the effect of the variable “year”. If there is no interannual variability, then the “year” should have a minor importance in the model. We have further clarified this fact in the new version of the manuscript (see line 346-349).

L325. You said in methods you used two periods of migration, instead you present here eight different periods. Can you group information for only those two period? More detailed results could be placed in the Supp Material and in the main text a general figure highlighting the detected stop-overs on the two different periods. Breeding and non-breeding known areas in the figure also would be very useful.

**Authors:** As it is stated in the Methods section (lines 190-196), actually we use two periods of migration (pre-breeding and post-breeding) so we built two specific models for one each other. However, also in the Methods section (lines 305-307), we indicate that “as an example of the potential marine areas that can be identified from the models, we predicted shearwater abundance across the study area on the fifteenth of the month, from May to December, based on the environmental conditions during 2017”, which are the eight different periods mentioned by the reviewer. As it is state in the text (lines 370-374), May to August represent the northward migration whereas September to December show the southward migration period. However, we have now explicitly indicated (see line 374 and new additions in the legend of the Figure 5) that May-August correspond to the post-breeding period and September-December to the pre-breeding. Therefore, we think that the information for both periods in Figure 5 is clearly differentiated.

Regarding the stop-overs, as it is highlighted in the Discussion section (see lines 436-437) , “our predictions should be taken rather as an illustration than as an exact calculation, since they are based on a single snapshot on the fifteenth of the month in a particular year”. An accurate estimation of stop-overs along the migratory route requires to quantify the distribution of abundance along the period in both seasons and apply appropriate methods for delimiting the marine areas (such as spatial prioritization algorithms). This work is part of the present research of the co-authors of the manuscript.

Regarding breeding and non-breeding areas, we think that adding these layers into Figure 5 would make hard to interpret the model results. However, this information has now been added in the new Figure 1.

L325. Figure suggests part of the population remains in the Mediterranean year-round. Is that true or it is a product of the modelling?

**Authors:** A variable fraction of the total Balearic shearwater population, mainly adult birds, appeared to remain in the Mediterranean (Ruiz and Martí, 2004). This is now discussed in the manuscript (see lines 497-500).

Ruiz, A., Martí, R., 2004. La Pardela Balear. SEO/BirdLife-Conselleria de Medi Ambient del Govern de les Illes Balears, Madrid, Spain.

L348-351. It is important to highlight that boosted regression trees methods such as the used here can artificially inflate accuracy with increasing iterations, therefore the need to evaluate how fit and accuracy varies with iterations. It is possible to have increasing accuracy and loss of fit to the point that the model starts predicting the response itself (occurrence or abundance) disregarding the factors, therefore one should use this in order to select the optimum number of iterations to be used in the final model outputs. This is not clear in methods, and this is not discussed either.

**Authors:** We think that the reviewer confuses “boosting” with “bagging” (used in Random Forest, see also comment above). As it is clearly stated in the manuscript (see lines ¿?), we do not apply boosted regression trees but Random Forest models. And any random forest model apply bagging (i.e., bootstrap aggregating) to sub-sample the data that are used for training. Random forests builds each tree independently while boosting builds one tree at a time and, while random forests combine results at the end of the process (by averaging or "majority rules"), boosting combines results along the way. For these reasons, boosting can result in better performance than random forests. However, in situations where a lot of noise is present (as it is our case), boosting may not be a good choice because it can result in overfitting (as the reviewer pointed out). Furthermore, Random Forest is an ensemble of decision trees. Although the single decision tree is very sensitive to data variations and it can easily overfit to noise in the data, when we add trees to the Random Forest then the tendency to overfitting decreases thanks to bagging and random feature selection. For all the previous, we found Random Forest as the best modelling approach for our datasets (see also Methods section on the assessment of other modelling techniques).

L386-390. Nowhere in your results there are estimated response curves. So please cite a reference to this statement.

**Authors:** See new Figures S4-S6 in the Supplementary material and line 442 in the Discussion section.

L389. It would be very useful to place in the map the sites you mention here, such as Alboran Sea or Gulf of Cadiz in figure 1, so readers not familiar with this region of the globe can be spatially situated.

**Authors:** Done.

L392-394. I thought results indicated differences on migratory routes between periods. Did I misunderstand?

**Authors:** In the Results section it is stated that, “according to the models, Balearic Shearwaters use slightly different regions during their northward (May–August, post-breeding) and southward migration (September–December, pre-breeding)”. We think that this is indeed not in contradiction with the above-mentioned sentence by the reviewer (“our results also suggested that Atlantic coast of Portugal and France are migratory, stopover and/or moulting sites for Balearic shearwaters both during pre and post-breeding migration”. As the regions are slightly different, Atlantic areas in Portugal and France are both used during pre- and post-breeding whereas other areas (also detailed in the Discussion section a few lines below) are clearly different for both migration periods (see lines 451-456: “the post-breeding model showed that Western English Channel is an important area for Balearic shearwaters mainly during August, whereas high abundance of shearwaters is predicted along the Algerian/Tunisian coastline in November”).

L397. Yet, how temporal variability was used in the model is not clear.

**Authors:** Temporal variability is explicitly modelled by “date” and “year” predictors, as well as, of course, indirectly modelled by the temporal variability in the environmental predictors. Please see lines 212-214 in the Methods section and also the Supplementary Methods in the Supplementary material describing the environmental variables modulating the migratory behaviour of shearwaters. See also comments above regarding the “effect to year” and the interaction between year and latitude in Figure 4.

L417. Again, without estimated response curves, I don’t think it is possible to reach such conclusion. A variable having high importance in the modelling doesn’t mean the birds had higher abundance in the higher values of the variable, this is particularly true using a model as RF that does not necessarily assumes linearity.

**Authors:** We have now added additional information in this regard in the Supplementary Material. Please see new Figures S4-S6.

L423. How variability in accuracy (that was not as large as the authors claimed) was led by the link with food availability? It is not clear. Needs better explanation.

**Authors:** As it is stated in the Results section, the difference in RMSE (i.e. model accuracy) between pre- and post-breeding models, even though small, is statistically significant at a Bonferroni p-level (see new addition in line 330 to highlight this point). If the distribution of the shearwater abundance depends on static predictors such as bathymetry, then the differences among resamples built with different training data sets are expected to be lower, thus the overall accuracy among resamples should be less variable. However, if migration progression depends on highly spatially and temporally variable predictors such as chlorophyll (food availability) then the variability in the accuracy among resamples is expected to be larger. We have now clarified this point in the text (see lines 481-485).

7. PLOS authors have the option to publish the peer review history of their article ([what does this mean?](https://journals.plos.org/plosone/s/editorial-and-peer-review-process#loc-peer-review-history)). If published, this will include your full peer review and any attached files.


If you choose “no”, your identity will remain anonymous but your review may still be made public.


**Do you want your identity to be public for this peer review?** For information about this choice, including consent withdrawal, please see our [Privacy Policy](https://www.plos.org/privacy-policy).

Reviewer #1: No

Reviewer #2: Yes: Lucas Krüger


[NOTE: If reviewer comments were submitted as an attachment file, they will be attached to this email and accessible via the submission site. Please log into your account, locate the manuscript record, and check for the action link "View Attachments". If this link does not appear, there are no attachment files to be viewed.]


While revising your submission, please upload your figure files to the Preflight Analysis and Conversion Engine (PACE) digital diagnostic tool, <https://pacev2.apexcovantage.com/>. PACE helps ensure that figures meet PLOS requirements. To use PACE, you must first register as a user. Registration is free. Then, login and navigate to the UPLOAD tab, where you will find detailed instructions on how to use the tool. If you encounter any issues or have any questions when using PACE, please email us at [figures@plos.org](mailto:figures@plos.org). Please note that Supporting Information files do not need this step.

*In compliance with data protection regulations, you may request that we remove your personal registration details at any time.* [*(Remove my information/details)*](https://www.editorialmanager.com/pone/login.asp?a=r)*. Please contact the publication office if you have any questions.*
